# Comparative Trends in the Burden of Depressive Disorders in China and Globally, 1990–2021: Evidence From the Global Burden of Disease 2021

**DOI:** 10.31083/AP48228

**Published:** 2026-06-29

**Authors:** Rui Li, Jiawen Huo, Junjiao Ping, Jing Wan, Xuan Ren, Shuyi Zhu, Aoxiang Luo, Tingyun Jiang

**Affiliations:** ^1^Department of Psychiatry, The Third People’s Hospital of Zhongshan, 528451 Zhongshan, Guangdong, China; ^2^Department of Scientific Research and Education, The Third People’s Hospital of Zhongshan, 528451 Zhongshan, Guangdong, China; ^3^Department of Medical Affairs, The Third People’s Hospital of Zhongshan, 528451 Zhongshan, Guangdong, China; ^4^School of Nursing, Guangdong Pharmaceutical University, 510310 Guangzhou, Guangdong, China

**Keywords:** depressive disorders, China, global health, global burden of disease, epidemiology, regression analysis

## Abstract

**Background:**

Depressive disorders are among the leading causes of global disability, with steadily increasing incidence and prevalence posing serious challenges to public health systems. As one of the most populous countries worldwide, China’s disease burden trajectory holds significant implications for global mental health trends. However, few studies have systematically compared China’s long-term trends with global patterns. This study aimed to evaluate the disease burden of depressive disorders in China from 1990 to 2021 using Global Burden of Disease Study 2021 (GBD 2021) data and to systematically compare these trends with global trajectories, with additional analysis across age and sex subgroups.

**Methods:**

We extracted data on the number of incident cases, prevalent cases, and disability-adjusted life years (DALYs) for depressive disorders, along with their corresponding age-standardized incidence rate (ASIR), age-standardized prevalence rate (ASPR), and age-standardized DALY rate (ASDR) from the GBD 2021 database. Joinpoint regression was used to estimate the estimated annual percentage change (EAPC) and the average annual percentage change (AAPC). The statistical significance of temporal trends was assessed based on 95% confidence intervals and two-sided *p *values (<0.05), with stratified analyses by age group and sex.

**Results:**

From 1990 to 2021, the number of depressive disorder cases in China showed an overall increasing trend, whereas ASIR, ASPR, and ASDR exhibited a gradual overall decline; in contrast, these age-standardized indicators showed a slight overall increase at the global level. Age-stratified analyses showed that ASIR, ASPR, and ASDR increased rapidly among individuals aged 10–24 years in both China and globally. From age 25 onward, the global burden of depressive disorders was mainly concentrated in the 25–49-year age group and declined with increasing age; in China, however, the disease burden remained at relatively high levels among middle-aged and older adults, with ASPR and ASDR peaking in the 40–69-year age group and then declining gradually. Sex-stratified analyses showed that, in both China and globally, females had higher overall disease burden levels than males in terms of incident cases, prevalent cases, DALYs, and corresponding age-standardized indicators.

**Conclusions:**

Despite China’s declining standardized burden, depressive disorders remain a major public health concern. The observed differences in age distribution and sex-specific burden, as well as divergent trends between China and global levels, underscore the need for targeted mental health interventions focused on adolescents, older adults, and women. These findings contribute to the evidence base for formulating population-specific mental health policies through a global comparative lens.

## Main Points

• Depressive disorder burden showed divergent trends between China and the global level from 1990 to 2021.

• China showed declining age-standardized rates despite an increasing number of depressive disorder cases.

• The burden increased rapidly among individuals aged 10–24 years in both China and globally.

• Middle-aged and older adults in China, and females in both China and globally, remained key high-burden populations.

## 1. Introduction

Depressive disorders are a group of common mental illnesses characterized by persistent low mood, loss of interest, and impaired daily functioning. According to the Global Burden of Disease Study 2019 (GBD 2019), mental disorders accounted for 1566.2 disability-adjusted life years (DALYs) per 100,000 population globally in 2019, with depressive disorders contributing up to 37.3% of that burden. Globally, mental disorders ranked seventh among the leading causes of DALYs in 2019, while depressive disorders ranked 13th among the top 25 leading causes of DALYs at the disorder level [1,2,3]. As one of the leading causes of non-fatal disability globally, depressive disorders significantly impair individuals’ quality of life and social functioning, posing an increasing challenge to public health systems and socioeconomic development [4].

Although pharmacological and psychological interventions have made substantial advances, a substantial treatment gap remains, with less than 50% of patients receiving adequate care globally [5]. Against the backdrop of growing international concern over mental health, China—one of the most populous developing countries—exhibits distinctive characteristics in terms of population size, rapid demographic transitions, and the ongoing development of its mental health service system; changes in the burden of depressive disorders in China therefore provide important reference value for understanding the global mental health landscape.

Although numerous studies have assessed the global burden of depressive disorders [2,6], the existing literature has largely focused on descriptive analyses at the global or regional level, often reporting temporal trends for the world as a whole or for individual countries separately [7]. Comparatively few studies have conducted systematic, long-term direct comparisons between specific countries and the global level within a unified analytical framework. This limitation has, to some extent, constrained a deeper understanding of the similarities and differences between the burden of depressive disorders in China and global trends, and has hindered accurate characterization of changes in China’s position within the global mental health landscape. In addition, relatively few studies have performed long-term comparative analyses of the structural differences in depressive disorder burden between China and the global population from demographic perspectives such as age and sex.

In recent years, the importance of global mental health issues has continued to rise. The World Health Organization (WHO), in its updated Comprehensive Mental Health Action Plan (2013–2030) [8] and the World Mental Health Report [9], has highlighted that depressive disorders have become a leading cause of disability worldwide. However, there are significant differences between countries in terms of the burden of depressive disorders, service coverage, and recovery capacity. Particularly after the COVID-19 pandemic, trends in the burden of depressive disorders have shown an imbalance, with some countries recovering quickly while others continue to face persistent service gaps. Against this international policy backdrop, analyzing the changes in depressive disorder burden in China—a rapidly developing country with a large population and swift social transformation—within the context of global trends provides valuable insight into the relative characteristics of China’s evolving mental health burden.

On this basis, rapid urbanization, structural social transitions, and the rise of digital technologies may exert both positive and negative impacts on mental health [10]. Meanwhile, China’s continuous efforts in health policy reform, expanded medical coverage, and the strengthening of public mental health interventions provide a distinctive model for disease control strategies [11,12]. In the context of increasingly prominent sex disparities and adolescent mental health risks, evaluating the evolving burden of disease across population subgroups can not only facilitate the identification of key intervention targets but also support the development of targeted mental health policies and data-driven decision-making [13,14].

Based on data from the GBD 2021 study, this research systematically analyzes the trends in incidence, prevalence, and DALYs for depressive disorders in China from 1990 to 2021, with a comparative perspective on global trends. It further explores the heterogeneity of disease burden across sex and age groups. Building upon prior research, this study extends the understanding of depressive burden in China through a longer time frame and more granular population stratification, enhancing its comparative significance within an international context. The findings of this study are intended to provide data support and theoretical reference for research in the field of mental health in China, and may also serve as a reference for other low- and middle-income countries in understanding the distribution patterns and temporal trends of mental health disease burden.

## 2. Materials and Methods

### 2.1 Data Sources and Indicator Selection

The data used in this study were obtained from the GBD 2021 [15], developed and maintained by the Institute for Health Metrics and Evaluation (IHME), University of Washington. The GBD database integrates information from multiple sources, including population surveys, hospital records, epidemiological studies, and death registries. It provides stratified estimates by country, sex, age group, and year across 204 countries and territories, enabling robust temporal and international comparability.

Data on depressive disorders were extracted from the GBD Results Tool [16], under the category “Cause of death and injury”. The selection filters were set as follows: location = “China” and “Global”, metric = “number” and “rate”, sex = “both”, “male”, and “female”, and years = “1990–2021”.

Six key indicators were selected to assess the burden of depressive disorders in China from 1990 to 2021 and for international comparison:

Incidence: Number of new cases of depressive disorders in a given period, reflecting the intensity and speed of disease spread.

Prevalence: Total number of existing cases in the population during a defined period, indicating the cumulative disease burden.

Age-Standardized Incidence Rate (ASIR): Incidence rate adjusted for population age structure, allowing comparisons across regions and time.

Age-Standardized Prevalence Rate (ASPR): Prevalence rate adjusted for age distribution, removing demographic confounding to reveal actual risk levels.

Disability-Adjusted Life Years (DALYs): A composite metric combining years lived with disability (YLDs) and years of life lost (YLLs), calculated as DALYs = YLDs + YLLs. As depressive disorders are non-fatal conditions, YLLs are negligible, making DALYs approximately equal to YLDs, which directly reflects the health burden attributable to disability [17].

Age-Standardized DALY Rate (ASDR): DALY rate adjusted for age structure, suitable for interpopulation and temporal comparisons, often used in international health policy evaluations.

Based on the aforementioned data, the GBD estimates are derived from multi-source data integration and statistical modeling methods, with the results being somewhat dependent on the availability and quality of the underlying data.

### 2.2 Statistical Analysis

#### 2.2.1 Descriptive Statistical Analysis

Descriptive statistical analysis and visualization were performed to evaluate the trends in incidence, prevalence, DALYs, and their age-standardized rates (ASIR, ASPR, ASDR) of depressive disorders among Chinese populations from 1990 to 2021. The percentage change was calculated using Eqn. 1 to describe temporal variations.


(1) $$Percentage\;Change=\frac{Value\;2021-Value\;1990}{Value\;1990}\times 100\%$$

To facilitate comparative analysis, the same indicators—including ASIR, ASPR, ASDR, and DALYs—were also extracted at the global level. Visual and statistical comparisons were performed to assess differences in temporal trends between China and the global average. Specifically, we compared percentage changes as well as slope estimates derived from Joinpoint regression analysis. In addition, stratified analyses by age and sex were conducted at both levels to explore population-specific disparities in depressive disorder burden.

#### 2.2.2 Joinpoint Regression Analysis

To further examine the time trends of key indicators, this study applied estimated annual percentage change (EAPC) and average annual percent change (AAPC), along with 95% confidence intervals (95% CI), to evaluate the direction and statistical significance of the trends. EAPC represents the model-based average annual percentage change in age-standardized rates over the full study period from 1990 to 2021, rather than the simple endpoint percentage change between 1990 and 2021.

Trend modeling and joinpoint estimation are used to identify statistically significant change points (joinpoints) in the time series and divide the overall trend into several linear phases. The annual percent change (APC) and its 95% CI are calculated for each phase. The reason for choosing the Joinpoint regression method is that it can identify statistically significant trend change points (joinpoints) in long-term time series data and provide segmented estimates of the rate of change across different time periods, making it suitable for long-term trend analysis of disease burden indicators [18,19]. Joinpoint regression analysis was performed based on age-standardized rates with log transformation, with the maximum number of joinpoints set to 5. The default model selection strategy of the Joinpoint Regression Program was applied, and the optimal number of joinpoints was determined through permutation testing.

If the lower boundary of the 95% CI of EAPC or AAPC was greater than 0, the trend was considered significantly increasing; if the upper boundary was less than 0, it was considered significantly decreasing. If the 95% CI included 0, the trend was considered not statistically significant [20,21]. A two-sided *p*-value < 0.05 was considered statistically significant.

Joinpoint regression analysis and its graphical plotting were performed using the Joinpoint Regression Program 5.1.0.0 (National Cancer Institute, Bethesda, MD, USA). Other data processing and graphical plotting were performed using Python 3.13 (Python Software Foundation, Beaverton, OR, USA).

## 3. Results

### 3.1 Temporal Trends in the Global and Chinese Burden of Depressive Disorders From 1990 to 2021

From 1990 to 2021, the global and Chinese incidence, prevalence, and burden of depressive disorders—as measured by DALYs—all showed an increasing trend. Compared with 1990, the global incidence, prevalence, and DALYs, incidence, and prevalence in 2021 increased by 93%, 89%, and 90%, respectively, while the corresponding increases in China were 39%, 54%, and 45%, indicating a lower growth rate in China than the global average (Table 1).

**Table 1.** **Comparative analysis of trends in depressive disorder incidence, prevalence, and DALYs between the global level and China, 1990–2021**.

Measure	Region	All-ages cases, thousands *n *(95% UI)		Percentage change	Age-standardized rates per 100,000 people (95% UI)		EAPC (95% CI)	AAPC (95% CI)
		1990	2021		1990	2021		
Incidence	Global	185,516.13 (162,602.51, 217,057.32)	357,438.73 (311,521.93, 418,969.49)	0.93	3748.50 (2869.53, 4862.59)	4333.64 (3253.18, 5707.15)	–0.06 (–0.22, 0.11)	0.49 (0.40, 0.63)
	China	30,491.04 (26,775.06, 35,440.16)	42,360.18 (36,991.76, 49,036.06)	0.39	2628.68 (2025.53, 3383.61)	2345.12 (1803.10, 3003.77)	–0.57 (–0.72, –0.42)	–0.35 (–0.42, –0.28)
Prevalence	Global	176,327.21 (158,931.45, 199,374.73)	332,410.33 (297,742.04, 376,102.44)	0.89	3599.67 (2925.58, 4396.05)	4006.83 (3215.08, 4963.82)	–0.03 (–0.15, 0.08)	0.36 (0.30, 0.45)
	China	34,479.39 (31,145.10, 38,431.73)	53,114.66 (47,435.18, 59,334.61)	0.54	3071.84 (2527.65, 3707.41)	2875.69 (2372.54, 3463.77)	–0.44 (–0.50, –0.38)	–0.21 (–0.24, –0.19)
DALYs	Global	29,674.67 (20,748.74, 40,205.91)	56,330.36 (39,339.99, 76,538.17)	0.90	600.52 (394.92, 856.37)	681.14 (442.81, 978.87)	–0.04 (–0.18, 0.10)	0.43 (0.35, 0.54)
	China	5426.67 (3756.63, 7330.30)	7865.94 (5560.59, 10,696.83)	0.45	473.32 (314.82, 670.26)	430.61 (288.37, 605.76)	–0.53 (–0.62, –0.43)	–0.28 (–0.32, –0.23)

Table note: UI, uncertainty interval; CI, confidence interval; EAPC, estimated annual percentage change; AAPC, average annual percentage change; DALYs, disability-adjusted life years. EAPC represents the model-based annual change from 1990 to 2021, not the endpoint change between 1990 and 2021. All numbers are presented in thousands unless otherwise specified. Percentage change is presented in decimal form (e.g., 0.93 represents a 93% change), multiplied by 100%.

**Fig. 1.**
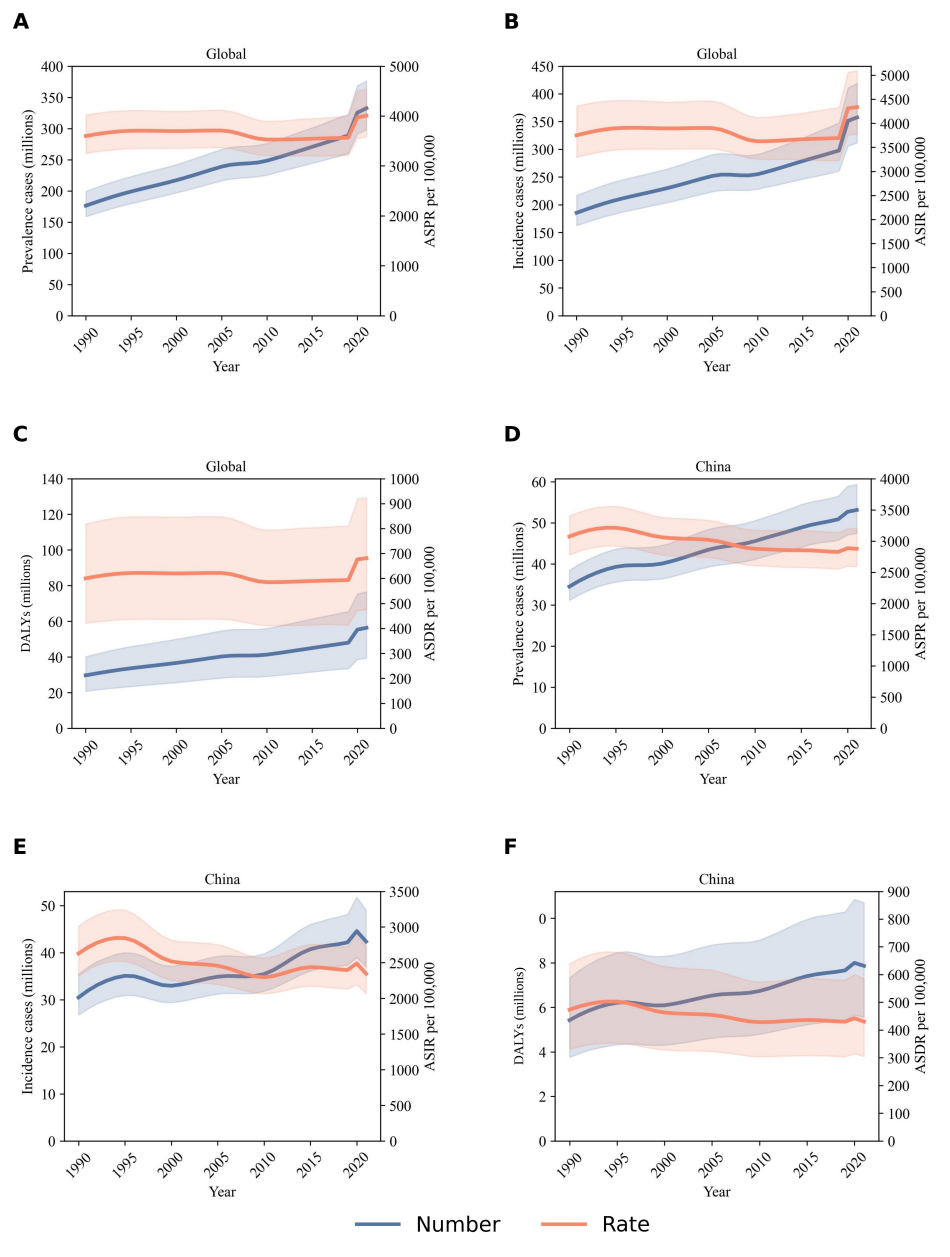
**Global and China trends in the burden of depressive disorders, 1990–2021**. (A) Global prevalence. (B) Global incidence. (C) Global DALYs. (D) China prevalence. (E) China incidence. (F) China DALYs, each shown from 1990 to 2021. In all panels, the blue line denotes the absolute number of cases (left y-axis, in millions), the red line denotes the age-standardized rate per 100,000 (right y-axis), and shaded bands show 95% UI from GBD 2021 estimates. Although x-axis tick labels are shown at 5-year intervals, the rightmost endpoint in each panel represents 2021. All rates are age-standardized to the GBD reference population. Abbreviations: GBD, Global Burden of Disease; ASPR, age-standardized prevalence rate; ASIR, age-standardized incidence rate; ASDR, age-standardized DALY rate; DALY, disability-adjusted life year; UI, uncertainty interval.

Fig. 1 illustrates the temporal trends in the case counts and age-standardized rates of depressive disorder incidence, prevalence, and DALYs between 1990 and 2021 in both the global and Chinese populations. Globally, the ASIR increased from 3748.50 per 100,000 population in 1990 to 4333.64 per 100,000 in 2021. The ASPR rose from 3599.67 to 4006.83 per 100,000, and the ASDR rose from 600.52 to 681.14 per 100,000. In contrast, China’s ASIR declined from 2628.68 to 2345.12 per 100,000, ASPR from 3071.84 to 2875.69 per 100,000, and ASDR from 473.32 to 430.61 per 100,000. The EAPCs of the three age-standardized indicators in China were –0.57%, –0.44%, and –0.53%, respectively, all showing a slow downward trend (Table 1).

**Fig. 2.**
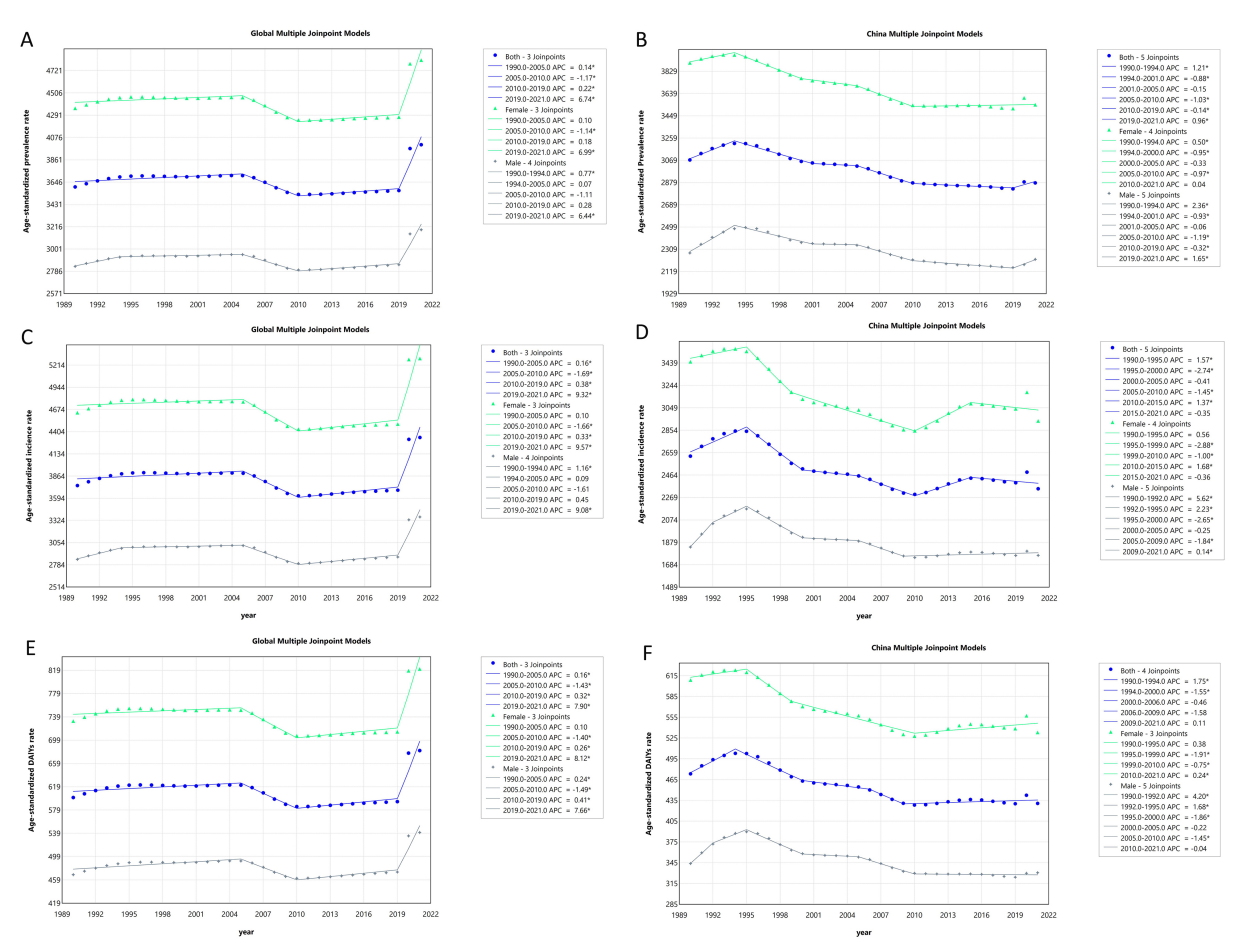
**Joinpoint regression analysis of the temporal trends in depressive disorder burden, 1990–2021**. (A) Global ASPR. (B) China ASPR. (C) Global ASIR. (D) China ASIR. (E) Global ASDR. (F) China ASDR, each analyzed using Joinpoint regression models to identify inflection points and corresponding APC segments. In each panel, blue, green, and gray lines represent both sexes, females, and males, respectively. Dots indicate observed values, and solid lines represent fitted regression trends. The number of joinpoints and APC values for each segment are shown on the right. *p* < 0.05 indicates a statistically significant APC. Abbreviations: ASPR, age-standardized prevalence rate; ASIR, age-standardized incidence rate; ASDR, age-standardized DALY rate; DALY, disability-adjusted life year; APC, annual percent change.

Fig. 2 presents the Joinpoint regression analysis of the ASIR, ASPR, and ASDR for depressive disorders in China and globally from 1990 to 2021; the corresponding Joinpoint regression parameters and sex-stratified AAPC values are provided in **Supplementary Table 1**. We observed that between 2005 and 2010, the global ASIR (APC = –1.69), ASPR (APC = –1.17), and ASDR (APC = –1.43) of depressive disorders showed a slow declining trend. However, the overall global trend was slightly upward, with AAPCs of 0.49%, 0.36%, and 0.43%, respectively.

In comparison, the ASIR in China exhibited a mild upward trend from 1990 to 1995 (APC = 1.57); the ASPR showed slow increases from 1990 to 1994 (APC = 1.21) and again from 2019 to 2021 (APC = 0.96). Similarly, the ASDR increased slightly from 1990 to 1994 (APC = 1.75) and from 2009 to 2021 (APC = 0.11).Overall, all three Chinese indicators demonstrated a modest downward trend over the study period, with AAPCs of –0.35%, –0.21%, and –0.28%, respectively.

During the COVID-19 pandemic period (2019–2021), the trend in the prevalence of depressive disorders (ASPR) showed significant differences compared to previous periods. Globally, the rate of increase in ASPR from 2019 to 2021 accelerated significantly (APC = 6.74), much higher than the slow increase observed during 2010–2019 (APC = 0.22) (see **Supplementary Table 2**). In China, ASPR exhibited a slight decline during 2010–2019 (APC = −0.14), but shifted to a slight increase in 2019–2021 (APC = 0.96), indicating a short-term trend reversal during the pandemic period (see **Supplementary Table 3**).

### 3.2 Age-Specific Patterns of Depressive Disorder Incidence, Prevalence, and Burden in 2021 in China and Globally

**Fig. 3.**
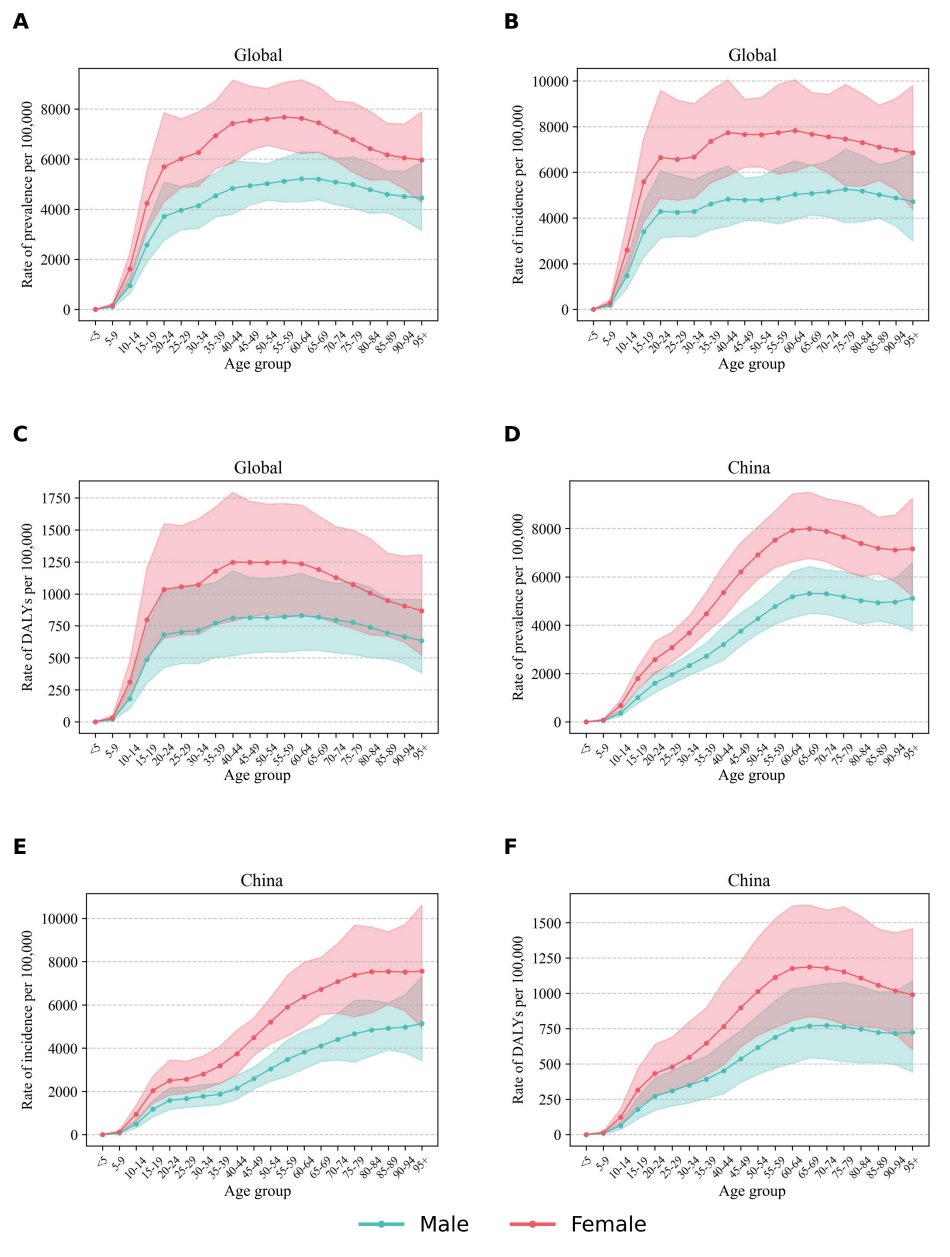
**Age- and sex-specific patterns of depressive disorder burden in 2021**. (A) Global ASPR. (B) Global ASIR. (C) Global ASDR. (D) China ASPR. (E) China ASIR. (F) China ASDR. Each panel presents the age-specific rate (per 100,000 population) of depressive disorders across 5-year age groups for males (blue line) and females (red line) in 1990–2021. Shaded areas represent the 95% UIs estimated by the Global Burden of Disease 2021 study. Abbreviations: ASPR, age-standardized prevalence rate; ASIR, age-standardized incidence rate; ASDR, age-standardized DALY rate; DALY, disability-adjusted life year; UI, uncertainty interval.

Fig. 3 illustrates the age-specific distribution of ASIR, ASPR, and ASDR for depressive disorders in 2021 in both China and globally (see **Supplementary Tables 4–6**). Both globally and in China, ASIR, ASPR, and ASDR increased rapidly between the ages of 10 and 24, indicating a substantial rise in depressive disorder burden during adolescence and early adulthood. After the age of 25, divergent trends emerged between the global and Chinese data. Globally, all three indicators peaked between the ages of 25 and 49 and subsequently declined, forming a pattern of middle-age concentration followed by attenuation in older age groups. In contrast, China’s ASIR continued to increase with age, remaining elevated in older age groups. ASPR and ASDR peaked between the ages of 40 and 69 and declined slowly thereafter, indicating a gradual rise during midlife and sustained burden into older adulthood.

**Fig. 4.**
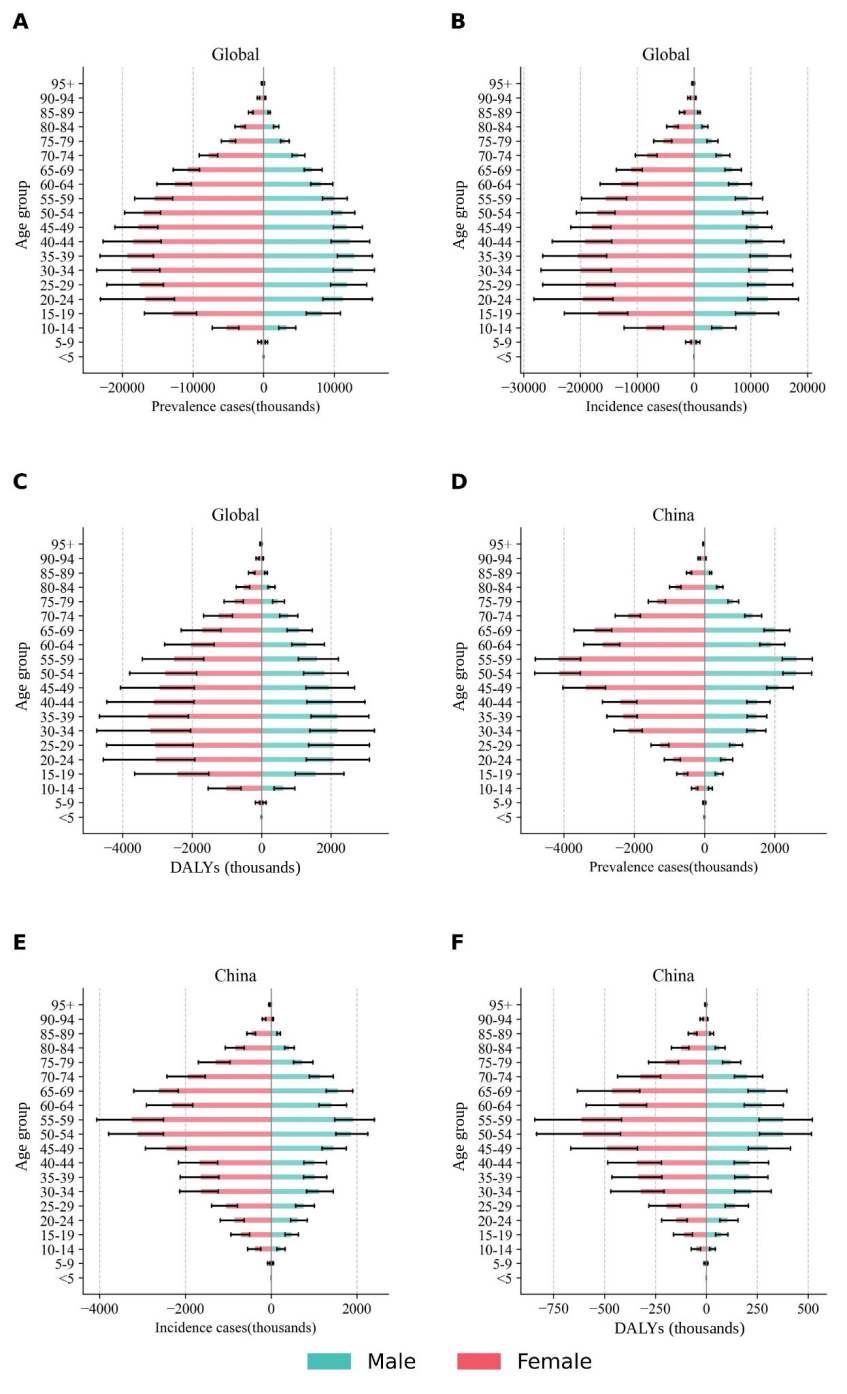
**Age- and sex-specific distribution of depressive disorder burden in 2021**. (A) Global prevalence cases. (B) Global incidence cases. (C) Global DALYs (thousands). (D) China prevalence cases. (E) China incidence cases. (F) China DALYs (thousands). Each panel illustrates the sex-specific distribution of depressive disorders across age groups in 2021. Pink bars represent females, and blue bars represent males. Values are shown as the number of cases (thousands) on a mirrored horizontal axis, with corresponding 95% UIs indicated by black error bars. The shape of the pyramids reflects the relative contribution of each age group to the total disease burden. All estimates were derived from the Global Burden of Disease 2021 study. Abbreviations: DALY, disability-adjusted life year; UI, uncertainty interval.

Fig. 4 further reflects these patterns in terms of absolute numbers of cases and overall disease burden. Globally, incident cases, prevalent cases, and DALYs were predominantly concentrated in the 20–49 age group, followed by a marked decline. In China, these indicators remained high between the ages of 45 and 69, with DALYs among individuals aged 60 years and older declining more slowly and remaining at a relatively high level throughout the older age range.

In 2021, the age distribution of depressive disorder burden in China showed a flatter and more delayed pattern compared to global trends, characterized by later peak burden and a more gradual post-peak decline.

### 3.3 Sex-Specific Trends in Depressive Disorder Burden in China, 1990–2021

**Fig. 5.**
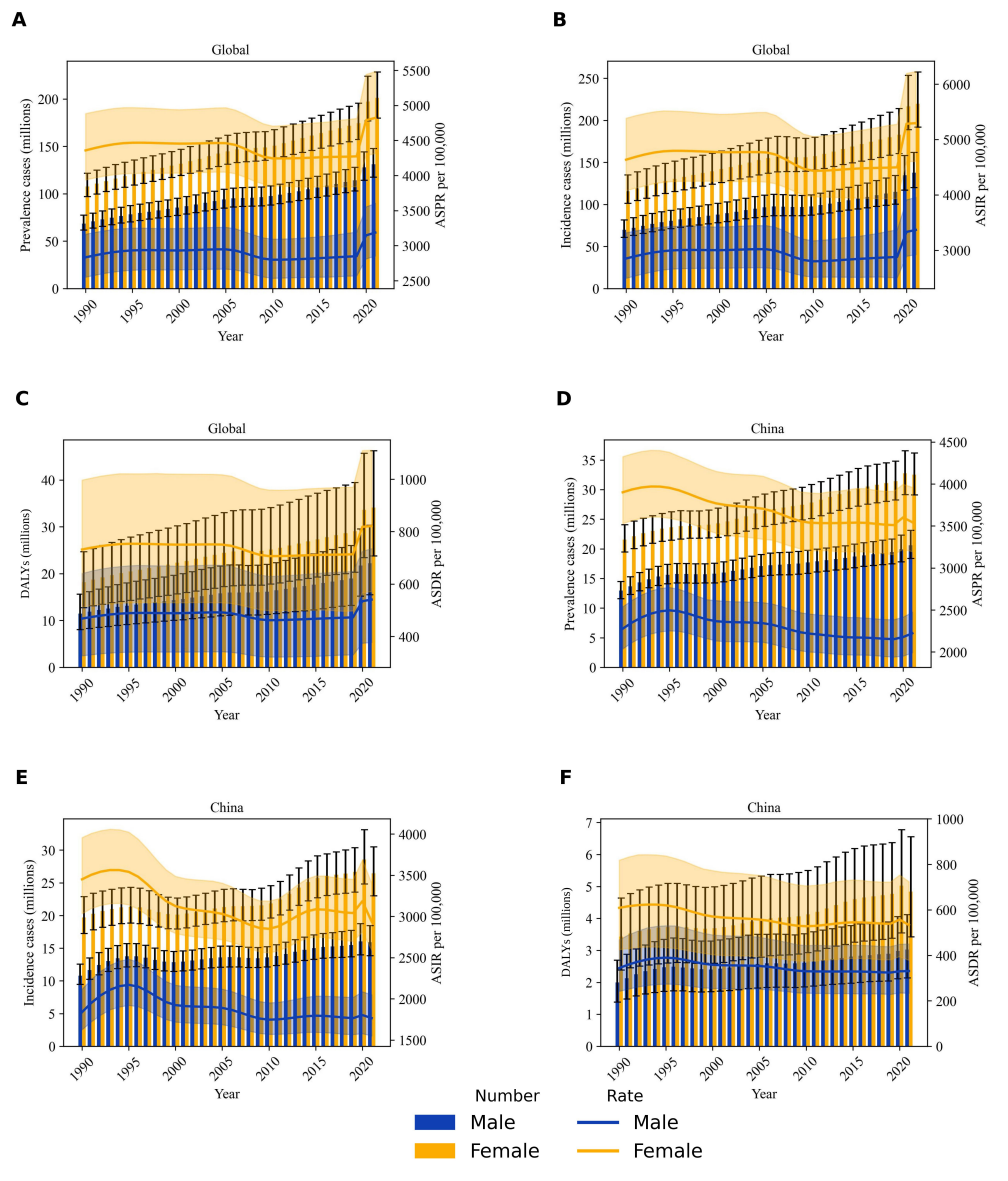
**Temporal trends in depressive disorder burden by sex, 1990–2021**. (A) Global prevalence. (B) Global incidence. (C) Global DALYs (millions). (D) China prevalence. (E) China incidence. (F) China DALYs (millions). Each panel illustrates the sex-specific temporal trends in depressive disorder burden from 1990 to 2021, showing both the absolute number of cases (left y-axis, in millions) and the age-standardized rate (right y-axis, per 100,000 population). Blue and orange lines represent males and females, respectively; bars indicate the corresponding number of cases. Shaded areas denote the 95% UIs estimated by the Global Burden of Disease 2021 study. Abbreviations: ASPR, age-standardized prevalence rate; ASIR, age-standardized incidence rate; ASDR, age-standardized DALY rate; DALY, disability-adjusted life year; UI, uncertainty interval.

Fig. 5 illustrates the trends in incidence, prevalence, and DALYs for depressive disorders among males and females in China and globally from 1990 to 2021, along with their corresponding age-standardized rates (ASIR, ASPR, and ASDR) (See **Supplementary Tables 7–12**). Throughout the observation period, females consistently exhibited higher values than males across all indicators, including incident cases, prevalent cases, DALYs, and age-standardized rates, at the overall level.

In addition, both male and female populations showed notable increases in ASIR, ASPR, ASDR, and absolute case counts from 2019 to 2020. Between 2020 and 2021, global figures for both sexes continued to rise modestly, whereas the corresponding indicators in China declined, with decreases observed in both crude numbers and age-standardized rates.

In terms of temporal trends, comparative analysis based on the AAPC showed that the trend changes in ASIR, ASPR, and ASDR were significantly greater in Chinese females than in males (95% confidence intervals did not overlap, *p* < 0.05). In contrast, the trend differences between males and females globally did not reach statistical significance (see **Supplementary Table 13**).

## 4. Discussion

Depressive disorder is one of the most prevalent mental health conditions worldwide and has long ranked among the leading contributors to the global burden of disease. This study reveals divergent temporal trends in the burden of depressive disorders between China and the global average from 1990 to 2021. While both China and the world have experienced increases in the absolute number of cases and total burden, China demonstrated a clear downward trend in age-standardized metrics, in contrast to the relatively stable or slightly increasing global patterns. The divergence is quantitatively supported by the AAPC results from Joinpoint regression: globally, the AAPCs for ASIR, ASPR, and ASDR were +0.49%, +0.36%, and +0.43%, respectively, whereas the corresponding values in China were −0.35%, −0.21%, and −0.28%, showing opposite directions of change. These findings further indicate that China and the global average exhibit increasingly distinct long-term trajectories in depressive disorder burden.

In recent years, China has made progress in the development of its mental health system, with mental health incorporated into the national basic public health service framework and with strengthened implementation of screening, diagnosis, and psychological interventions [22]. Meanwhile, research on depressive disorders in China has gradually shifted toward investigations of underlying etiological mechanisms, and the standardized use of antidepressant medications [23,24,25] together with the development of novel therapeutic approaches [26,27] has provided technical support for disease management. Although similar advances have also been observed globally, overall trends have remained relatively stable, with no clear decline. This global “plateau” phenomenon may be related to higher levels of disease recognition and broader service coverage [5], although the specific roles of these factors were not examined in the present study. By contrast, China has exhibited a more pronounced downward trend in the burden of depressive disorders, the underlying reasons for which require further investigation. Both China and the global population experienced a short-term increase in depressive disorder burden around 2020, which may be associated with the COVID-19 pandemic; differences in post-pandemic recovery trajectories further reflect variation in recovery capacity and the organization of mental health services across countries [28,29].

From an age-specific perspective, both globally and in China, the ASIR, ASPR, and ASDR of depressive disorders exhibit a pronounced upward trend during adolescence and young adulthood (ages 10–24). This pattern is consistent with biological factors occurring during this developmental stage, such as hormonal fluctuations [30] and neurodevelopmental changes in brain structure and function [31,32]. Meanwhile, adolescents are commonly exposed to increasing academic pressure, more intensive peer interactions, and rising family expectations [33], and these psychosocial factors may, to some extent, influence their emotional well-being. These trends suggest that adolescence and early young adulthood exhibit notable upward patterns in the age distribution of depressive disorder burden that warrant attention.

In terms of the age distribution of disease burden, the global burden of depressive disorders is primarily concentrated in individuals aged 20–49 years, whereas in China, it is predominantly concentrated in those aged 40–69 years. Although some indicators show a gradual decline in the older age groups, middle-aged and older adults still bear a high absolute disease burden and accumulated disability burden. This structural difference in burden may be influenced by a variety of factors, including demographic age composition, chronic disease comorbidity, cultural role expectations, and patterns of healthcare utilization. Globally, individuals aged 20–49 are typically at a life stage where economic and family responsibilities converge, often facing challenges such as occupational stress, fast-paced lifestyles, and shifting social roles [34,35,36,37,38]. By contrast, in China, middle-aged and older adults account for a relatively higher proportion of the burden of depressive disorders; this pattern may co-occur with several contextual factors, including accelerated population aging, a higher prevalence of chronic comorbidities, reduced social participation, changes in family roles, and culturally influenced ways of expressing psychological distress. Studies have shown that older adults have higher comorbidity rates of depressive disorders [39], and that changes in social support systems may be associated with increased psychological vulnerability in this population [40,41]. Additionally, under traditional cultural norms, older adults in China tend to conceal emotional problems or delay seeking treatment [42], and this behavioral pattern may be associated with the prolonged persistence of psychological distress.

In the context of China, a developing country with a large population and rapid social transformation, the differences in the burden of depressive disorders across age groups may reflect the combined effects of generational role changes and socio-cultural norms. Previous studies have commonly regarded highly competitive educational environments [43,44], the earlier onset of employment-related pressures [45,46], and social comparison in a digital society [47,48] as prevalent psychosocial contextual characteristics among adolescents and individuals in early young adulthood. In contrast, middle-aged and older adults are more likely to experience long-term psychological distress due to accelerating aging, increasing comorbid chronic diseases, and reduced social participation [49]; additionally, in the context of traditional culture, the recognition and intervention of mental health issues among middle-aged and older adults are often delayed relative to the actual onset of the disease [50]. As a populous country, China’s depressive disorder burden patterns shaped by the aggregation of different age groups provide an important case for understanding age-specific differences in depressive disorder burden from an international comparative perspective.

From 1990 to 2021, both globally and in China, females consistently exhibited higher overall disease burden levels in terms of incidence, prevalence, DALYs, and age-standardized rates compared to males, suggesting that sex-specific biological, psychological, and socio-cultural factors may play significant roles in the onset and development of depressive disorders. Biologically, women undergo substantial hormonal fluctuations during puberty, premenstrual periods, pregnancy, postpartum, perimenopause, and menopause, affecting neurotransmitter function and emotional regulation, and thereby increasing vulnerability to depressive disorders [51,52]. Meta-analyses of large-scale epidemiological studies also show thatwomen have nearly twice the lifetime risk of depression compared to men [53].

Psychologically, women are generally more expressive of their emotions and more likely to seek help, a characteristic that is associated with higher diagnostic rates; whereas men tend to suppress emotional distress due to stigma and sex norms, which may, to some extent, be related to underdiagnosis and delays in the treatment of depressive disorders [54,55]. In addition, male depressive symptoms are often manifested in atypical forms such as substance abuse, suicidal behavior, or aggression, which frequently do not align with traditional diagnostic criteria for depression (e.g., low mood, helplessness) [56,57]. Under the influence of the psychological and behavioral characteristics described above, depressive disorders among men may be insufficiently recognized in clinical diagnosis, which may, to some extent, lead to an underestimation of their disease burden.

In terms of socio-cultural influences, women frequently shoulder multiple roles in both family and workplace settings, including caregiving, parenting, professional responsibilities, and invisible labor, which cumulatively increase psychological stress [58,59]. Additionally, they are at heightened risk of experiencing traumatic events such as sexual and intimate partner violence [60], and a large body of evidence has established strong associations between these experiences and depressive disorders [61,62,63]. Taken together, these multidimensional burdens across the lifespan highlight the persistent sex-based imbalance in depression risk and disease burden.

From a public health perspective, this study, based on age- and sex-stratified analyses within a China–global comparative framework, reveals long-term differences in the evolution of depressive disorder burden across population groups. Adolescents and middle-aged to older adults exhibit burden trajectories that differ from those of other age groups, while sex differences remain consistent across multiple indicators, providing specific context for identifying patterns of differentiation in depressive disorder burden at the population level. When considered alongside time-series changes before and after the COVID-19 pandemic, these results help to characterize the dynamic evolution of depressive disorder burden and its cross-population heterogeneity in the context of public health emergencies.

This study has several strengths. It systematically analyzed the long-term trends in the burden of depressive disorders in China and globally from 1990 to 2021, incorporating incidence, prevalence, DALYs, and their age-standardized rates. By comparing across sex and age groups, the study presents heterogeneous patterns in the evolution of disease burden. In addition, this study conducted a comparative analysis of the trajectories of depressive disorder burden in China and globally around the critical time point of the COVID-19 pandemic. These analyses provide supplementary information for understanding the evolutionary patterns of depressive disorder burden across countries, particularly in low- and middle-income countries, from an international comparative perspective.

However, some limitations should be acknowledged. The inclusion of Chinese data in global estimates may have led to an underestimation of the differences between China and the global level. Additionally, part of the GBD data is based on model estimates rather than original sources, with reliability affected by regional data availability. Particularly in the elderly population and rural areas in China, the low reporting of depressive disorders may lead to potential underestimation in the model estimates. Moreover, as this study is descriptive in nature and lacks statistical tests or control for confounding variables, causal inferences regarding intervention effects cannot be drawn. Despite these limitations, the temporal trends and population distribution patterns identified in this study still provide important data support for future research on changes in the burden of depressive disorders.

## 5. Conclusions

This study systematically analyzes the long-term trends in the burden of depressive disorders in China and globally from 1990 to 2021, and compares the structural differences across age and sex dimensions. It also explores the changes in the disease burden evolution patterns before and after the COVID-19 pandemic. The results show that the ASIR, ASPR, and ASDR for depressive disorders in China generally declined from 1990 to 2021, with a divergence in the trend during the post-pandemic phase compared to the global trend. Age- and sex-stratified analyses revealed significant structural differences in the disease burden across different populations: globally and in China, the burden of depressive disorders was higher among adolescents, while the burden was most prominent in the young and middle-aged populations globally; in China, the peak burden for middle-aged and older adults occurred later and remained high for a longer period. Additionally, in terms of sex, females consistently had a higher disease burden than males. Overall, from a global comparative perspective, this study delineates the long-term patterns of change in the burden of depressive disorders in China and their population structural differences, offering useful insights for identifying priority populations and optimizing mental health service systems, while also providing valuable reference data for future cross-national comparative research.

## Data Availability

All data used in this study were obtained from the Global Burden of Disease 2021 database (Institute for Health Metrics and Evaluation, Seattle, WA, USA). The datasets analyzed are publicly available at https://vizhub.healthdata.org/gbd-results/. Processed data and analysis scripts are available from the corresponding author upon reasonable request.

## Abbreviations

GBD, Global Burden of Disease; DALY, disability-adjusted life year; UI, uncertainty interval; ASPR, age-standardized prevalence rate; ASIR, age-standardized incidence rate; ASDR, age-standardized DALY rate; EAPC, estimated annual percentage change.
