# Supplementary material for: Comparative Trends in the Burden of Depressive Disorders in China and Globally, 1990–2021: Evidence From the Global Burden of Disease 2021
Source: Alpha Psychiatry. 2026 Jun 29;27(3):48228. doi: 10.31083/AP48228 (PMC13339878; doi:10.31083/AP48228)
Supplement: Supplementary file 1 [file 2757-8038-27-3-48228-s1.zip › Supplementary Tables.docx]

***Supplementary Material***

**Supplementary Tables**

**Supplementary Table 1. Joinpoint regression results of sex-specific trends in depressive disorder burden, 1990–2021**

| Location | Measures | Cohort | Range | Lower Endpoint | Upper Endpoint | AAPC | Lower CI | Upper CI | *P*-Value |
| --- | --- | --- | --- | --- | --- | --- | --- | --- | --- |
| Global | Prevalence | Both - 3 Joinpoints | Full Range | 1990 | 2021 | 0.361 | 0.299 | 0.454 | < 0.001 |
| Global | Prevalence | Female - 3 Joinpoints | Full Range | 1990 | 2021 | 0.350 | 0.284 | 0.450 | < 0.001 |
| Global | Prevalence | Male - 4 Joinpoints | Full Range | 1990 | 2021 | 0.428 | 0.358 | 0.512 | < 0.001 |
| Global | Dalys | Both - 3 Joinpoints | Full Range | 1990 | 2021 | 0.426 | 0.348 | 0.541 | < 0.001 |
| Global | Dalys | Female - 3 Joinpoints | Full Range | 1990 | 2021 | 0.401 | 0.320 | 0.526 | < 0.001 |
| Global | Dalys | Male - 3 Joinpoints | Full Range | 1990 | 2021 | 0.469 | 0.395 | 0.573 | < 0.001 |
| Global | Incidence | Both - 3 Joinpoints | Full Range | 1990 | 2021 | 0.491 | 0.397 | 0.630 | < 0.001 |
| Global | Incidence | Female - 3 Joinpoints | Full Range | 1990 | 2021 | 0.466 | 0.366 | 0.614 | < 0.001 |
| Global | Incidence | Male - 4 Joinpoints | Full Range | 1990 | 2021 | 0.614 | 0.508 | 0.743 | < 0.001 |
| China | Prevalence | Both - 5 Joinpoints | Full Range | 1990 | 2021 | -0.210* | -0.238 | -0.186 | < 0.001 |
| China | Prevalence | Female - 4 Joinpoints | Full Range | 1990 | 2021 | -0.315* | -0.345 | -0.288 | < 0.001 |
| China | Prevalence | Male - 5 Joinpoints | Full Range | 1990 | 2021 | -0.100* | -0.144 | -0.067 | < 0.001 |
| China | Incidence | Both - 5 Joinpoints | Full Range | 1990 | 2021 | -0.348* | -0.417 | -0.279 | < 0.001 |
| China | Incidence | Female - 4 Joinpoints | Full Range | 1990 | 2021 | -0.446* | -0.541 | -0.354 | < 0.001 |
| China | Incidence | Male - 5 Joinpoints | Full Range | 1990 | 2021 | -0.093* | -0.141 | -0.031 | 0.003 |
| China | Dalys | Both - 4 Joinpoints | Full Range | 1990 | 2021 | -0.280* | -0.323 | -0.234 | < 0.001 |
| China | Dalys | Female - 3 Joinpoints | Full Range | 1990 | 2021 | -0.368* | -0.426 | -0.297 | < 0.001 |
| China | Dalys | Male - 5 Joinpoints | Full Range | 1990 | 2021 | -0.160* | -0.190 | -0.124 | < 0.001 |

**Supplementary Table 2. Joinpoint regression results for sex-specific segmented trends in depressive disorder prevalence, Global, 1990–2021 (APC by segment)**

| Cohort | Segment | Lower Endpoint | Upper Endpoint | APC | Lower CI | Upper CI | *P*-Value |
| --- | --- | --- | --- | --- | --- | --- | --- |
| Both - 3 Joinpoints | 1 | 1990 | 2005 | 0.14* | 0.04 | 0.26 | 0.009 |
| Both - 3 Joinpoints | 2 | 2005 | 2010 | -1.17* | -2 | -0.73 | < 0.001 |
| Both - 3 Joinpoints | 3 | 2010 | 2019 | 0.22* | 0.04 | 0.45 | 0.016 |
| Both - 3 Joinpoints | 4 | 2019 | 2021 | 6.74* | 4.41 | 8.3 | < 0.001 |
| Female - 3 Joinpoints | 1 | 1990 | 2005 | 0.1 | 0 | 0.24 | 0.053 |
| Female - 3 Joinpoints | 2 | 2005 | 2010 | -1.14* | -1.98 | -0.66 | < 0.001 |
| Female - 3 Joinpoints | 3 | 2010 | 2019 | 0.18 | 0 | 0.44 | 0.053 |
| Female - 3 Joinpoints | 4 | 2019 | 2021 | 6.99* | 4.56 | 8.69 | < 0.001 |
| Male - 4 Joinpoints | 1 | 1990 | 1994 | 0.77* | 0.19 | 1.75 | 0.006 |
| Male - 4 Joinpoints | 2 | 1994 | 2005 | 0.07 | -1.11 | 0.23 | 0.551 |
| Male - 4 Joinpoints | 3 | 2005 | 2010 | -1.11 | -1.84 | 0.71 | 0.0863 |
| Male - 4 Joinpoints | 4 | 2010 | 2019 | 0.28 | -0.13 | 0.48 | 0.070 |
| Male - 4 Joinpoints | 5 | 2019 | 2021 | 6.44* | 4.21 | 7.89 | < 0.001 |

**Supplementary Table 3. Joinpoint regression results for sex-specific segmented trends in depressive disorder prevalence, China, 1990–2021 (APC by segment)**

| Cohort | Segment | Lower Endpoint | Upper Endpoint | APC | Lower CI | Upper CI | *P*-Value |
| --- | --- | --- | --- | --- | --- | --- | --- |
| Both - 5 Joinpoints | 1 | 1990 | 1994 | 1.21* | 1.04 | 1.39 | < 0.001 |
| Both - 5 Joinpoints | 2 | 1994 | 2001 | -0.88* | -1.02 | -0.79 | < 0.001 |
| Both - 5 Joinpoints | 3 | 2001 | 2005 | -0.15 | -0.39 | 0.14 | 0.269 |
| Both - 5 Joinpoints | 4 | 2005 | 2010 | -1.03* | -1.36 | -0.87 | < 0.001 |
| Both - 5 Joinpoints | 5 | 2010 | 2019 | -0.14* | -0.25 | -0.06 | 0.004 |
| Both - 5 Joinpoints | 6 | 2019 | 2021 | 0.96* | 0.38 | 1.32 | < 0.001 |
| Female - 4 Joinpoints | 1 | 1990 | 1994 | 0.50* | 0.18 | 1.07 | 0.004 |
| Female - 4 Joinpoints | 2 | 1994 | 2000 | -0.95* | -1.43 | -0.78 | < 0.001 |
| Female - 4 Joinpoints | 3 | 2000 | 2005 | -0.33 | -0.54 | 0.1 | 0.136 |
| Female - 4 Joinpoints | 4 | 2005 | 2010 | -0.97* | -1.39 | -0.73 | 0.018 |
| Female - 4 Joinpoints | 5 | 2010 | 2021 | 0.04 | -0.04 | 0.13 | 0.235 |
| Male - 5 Joinpoints | 1 | 1990 | 1994 | 2.36* | 2.12 | 2.64 | < 0.001 |
| Male - 5 Joinpoints | 2 | 1994 | 2001 | -0.93* | -1.2 | -0.82 | < 0.001 |
| Male - 5 Joinpoints | 3 | 2001 | 2005 | -0.06 | -0.39 | 0.33 | 0.668 |
| Male - 5 Joinpoints | 4 | 2005 | 2010 | -1.19* | -1.7 | -0.96 | < 0.001 |
| Male - 5 Joinpoints | 5 | 2010 | 2019 | -0.32* | -0.45 | -0.18 | 0.002 |
| Male - 5 Joinpoints | 6 | 2019 | 2021 | 1.65* | 0.8 | 2.09 | < 0.001 |

**Supplementary Table 4. Incidence of depressive disorders by age group (Both sexes, Global and China, 1990–2021)**

| Location | Age  (years) | 1990,number(thousands)  (95% UI) | 2021,number(thousands)  (95% UI) | percentage change  (100%) | 1990_per100000  (95% UI) | 2021_per100000  (95% UI) | EAPC  (95% CI) |
| --- | --- | --- | --- | --- | --- | --- | --- |
| Global | <5 | 15.51(8.06,25.43) | 23.18(11.67,39.31) | 0.49 | 2.5(1.3,4.1) | 3.52(1.77,5.97) | 0.53 (0.31, 0.76) |
| Global | 5-9 | 942.23(514.95,1501.65) | 1568.36(818.60,2514.87) | 0.66 | 161.47(88.25,257.34) | 228.27(119.15,366.04) | 0.73 (0.53, 0.94) |
| Global | 10-14 | 7496.23(4732.11,10869.04) | 13424.11(8546.54,19698.77) | 0.79 | 1399.38(883.38,2029.01) | 2013.71(1282.04,2954.95) | 0.74 (0.54, 0.94) |
| Global | 15-19 | 17929.06(12623.49,24345.05) | 27848.83(19026.21,37536.13) | 0.55 | 3451.72(2430.29,4686.94) | 4463.1(3049.17,6015.6) | 0.17 (-0.05, 0.39) |
| Global | 20-24 | 22313.75(16624.93,30759.01) | 32536.74(23813.25,46626.26) | 0.46 | 4534.5(3378.45,6250.71) | 5448.6(3987.76,7808.03) | -0.18 (-0.43, 0.08) |
| Global | 25-29 | 20568.20(15673.53,27681.12) | 31750.57(23337.59,43962.73) | 0.54 | 4646.92(3541.08,6253.93) | 5396.61(3966.66,7472.29) | -0.24 (-0.49, 0.00) |
| Global | 30-34 | 18648.36(13919.76,24339.56) | 33039.19(24204.71,44343.77) | 0.77 | 4838.42(3611.56,6315.03) | 5465.72(4004.22,7335.85) | -0.24 (-0.44, -0.04) |
| Global | 35-39 | 17953.85(14011.77,22988.44) | 33520.81(25340.04,43610.65) | 0.87 | 5096.99(3977.85,6526.27) | 5976.62(4518.02,7775.59) | -0.11 (-0.32, 0.09) |
| Global | 40-44 | 15341.35(11813.74,19866.55) | 31365.86(23656.06,40953.71) | 1.04 | 5355.09(4123.73,6934.67) | 6270.03(4728.84,8186.64) | -0.12 (-0.32, 0.09) |
| Global | 45-49 | 12787.10(10423.85,15406.51) | 29456.09(23956.05,35310.54) | 1.30 | 5507.04(4489.26,6635.14) | 6220.87(5059.31,7457.27) | -0.14 (-0.30, 0.02) |
| Global | 50-54 | 11846.19(9716.92,14258.65) | 27675.50(22517.95,33495.86) | 1.34 | 5572.8(4571.13,6707.7) | 6220.29(5061.09,7528.46) | -0.10 (-0.22, 0.03) |
| Global | 55-59 | 10436.56(8109.22,13338.49) | 25028.53(19231.20,31746.04) | 1.40 | 5635.29(4378.63,7202.21) | 6324.68(4859.7,8022.19) | -0.04 (-0.17, 0.08) |
| Global | 60-64 | 9297.57(7241.56,11830.02) | 20721.95(16130.11,26572.82) | 1.23 | 5788.94(4508.81,7365.71) | 6474.64(5039.91,8302.76) | -0.02 (-0.14, 0.10) |
| Global | 65-69 | 7312.09(6029.46,9015.02) | 17745.60(14605.47,21952.29) | 1.43 | 5915.48(4877.83,7293.14) | 6433.24(5294.87,7958.28) | 0.02 (-0.06, 0.10) |
| Global | 70-74 | 5132.48(4068.01,6403.00) | 13227.05(10404.34,16558.55) | 1.58 | 6062.38(4805.05,7563.09) | 6425.92(5054.6,8044.41) | 0.07 (0.03, 0.10) |
| Global | 75-79 | 3838.88(2814.01,5034.45) | 8525.22(6183.84,11312.06) | 1.22 | 6236.46(4571.5,8178.71) | 6464.16(4688.83,8577.25) | 0.10 (0.06, 0.13) |
| Global | 80-84 | 2274.35(1705.09,2882.19) | 5622.59(4145.06,7287.99) | 1.47 | 6429.09(4819.92,8147.31) | 6419.73(4732.72,8321.23) | 0.03 (-0.03, 0.09) |
| Global | 85-89 | 1012.64(816.41,1259.82) | 2890.67(2299.13,3638.77) | 1.85 | 6701.3(5402.74,8337.04) | 6322.31(5028.51,7958.51) | -0.14 (-0.21, -0.08) |
| Global | 90-94 | 296.93(225.23,394.14) | 1126.41(845.08,1492.48) | 2.79 | 6929.25(5255.93,9197.74) | 6296.51(4723.9,8342.82) | -0.26 (-0.32, -0.20) |
| Global | 95+ | 72.78(48.45,103.75) | 341.47(217.55,489.75) | 3.69 | 7149.17(4758.51,10190.67) | 6265.14(3991.58,8985.67) | -0.33 (-0.40, -0.27) |
| China | <5 | 2.14(1.06,3.51) | 1.78(0.89,2.94) | -0.17 | 1.92(0.95,3.14) | 2.29(1.15,3.78) | 0.36 (0.24, 0.48) |
| China | 5-9 | 95.08(52.98,150.47) | 87.18(48.55,136.04) | -0.08 | 91.18(50.81,144.3) | 91.03(50.69,142.05) | 0.36 (0.15, 0.56) |
| China | 10-14 | 888.07(568.08,1264.92) | 601.37(385.80,870.75) | -0.32 | 868.16(555.34,1236.58) | 697.7(447.59,1010.23) | -0.29 (-0.55, -0.04) |
| China | 15-19 | 3121.44(2225.14,4285.66) | 1175.88(827.63,1567.91) | -0.62 | 2464.32(1756.71,3383.46) | 1574.73(1108.35,2099.72) | -1.56 (-1.68, -1.44) |
| China | 20-24 | 4480.65(3353.17,6106.92) | 1469.79(1087.65,2038.63) | -0.67 | 3394.41(2540.26,4626.42) | 2008.62(1486.39,2785.99) | -2.11 (-2.35, -1.87) |
| China | 25-29 | 3711.33(2836.96,4820.03) | 1810.92(1365.79,2395.61) | -0.51 | 3377.33(2581.65,4386.25) | 2093.98(1579.27,2770.07) | -1.88 (-2.15, -1.61) |
| China | 30-34 | 2999.19(2257.98,3875.01) | 2742.88(2078.41,3572.76) | -0.09 | 3398.74(2558.78,4391.22) | 2263.98(1715.52,2948.96) | -1.63 (-1.90, -1.36) |
| China | 35-39 | 3253.36(2555.73,4058.11) | 2658.08(1996.87,3405.40) | -0.18 | 3561.85(2798.06,4442.91) | 2508.49(1884.49,3213.75) | -1.43 (-1.66, -1.19) |
| China | 40-44 | 2435.44(1880.56,3111.60) | 2677.50(2029.26,3450.71) | 0.10 | 3629.87(2802.86,4637.64) | 2925.15(2216.95,3769.88) | -0.98 (-1.19, -0.78) |
| China | 45-49 | 1870.43(1546.17,2263.91) | 3888.30(3182.37,4682.07) | 1.08 | 3623.54(2995.35,4385.82) | 3524.51(2884.63,4244.02) | -0.40 (-0.57, -0.22) |
| China | 50-54 | 1734.18(1428.17,2070.70) | 4969.04(4021.16,6030.55) | 1.87 | 3634.78(2993.39,4340.09) | 4111.44(3327.15,4989.74) | 0.07 (-0.09, 0.24) |
| China | 55-59 | 1586.66(1233.69,2007.88) | 5159.73(3998.92,6428.38) | 2.25 | 3658.49(2844.62,4629.75) | 4693.13(3637.29,5847.05) | 0.44 (0.28, 0.60) |
| China | 60-64 | 1351.30(1057.74,1743.86) | 3719.81(2964.01,4687.71) | 1.75 | 3823.99(2993.24,4934.88) | 5095.27(4060.0,6421.07) | 0.63 (0.47, 0.80) |
| China | 65-69 | 1141.34(941.10,1396.68) | 4167.00(3438.35,5099.91) | 2.65 | 4183.53(3449.56,5119.46) | 5432.61(4482.66,6648.86) | 0.67 (0.53, 0.82) |
| China | 70-74 | 854.66(676.74,1072.26) | 3081.17(2433.97,3876.63) | 2.61 | 4541.82(3596.33,5698.15) | 5781.19(4566.85,7273.71) | 0.69 (0.55, 0.82) |
| China | 75-79 | 557.83(413.39,745.10) | 2020.73(1483.94,2676.13) | 2.62 | 4901.58(3632.37,6547.01) | 6101.42(4480.65,8080.36) | 0.70 (0.57, 0.84) |
| China | 80-84 | 284.29(221.37,365.28) | 1256.66(947.56,1606.75) | 3.42 | 5366.88(4179.08,6895.78) | 6349.4(4787.61,8118.22) | 0.63 (0.51, 0.74) |
| China | 85-89 | 100.51(82.01,124.36) | 627.11(499.04,778.88) | 5.24 | 5958.52(4861.73,7372.26) | 6583.36(5238.86,8176.58) | 0.44 (0.36, 0.52) |
| China | 90-94 | 20.22(15.55,26.48) | 199.87(152.37,258.33) | 8.88 | 6590.16(5067.99,8629.09) | 6816.92(5196.92,8810.76) | 0.26 (0.20, 0.31) |
| China | 95+ | 2.89(2.01,4.09) | 45.38(29.80,63.91) | 14.68 | 7146.78(4966.69,10100.68) | 7100.41(4663.35,10000.29) | 0.12 (0.08, 0.17) |

**Supplementary Table 5. Prevalence of depressive disorders by age group (Both sexes, Global and China, 1990–2021)**

| Location | Age  (years) | 1990,number(thousands)  (95% UI) | 2021,number(thousands)  (95% UI) | percentage change(100%) | 1990_per100000  (95% UI) | 2021_per100000  (95% UI) | EAPC(95% CI) |
| --- | --- | --- | --- | --- | --- | --- | --- |
| Global | <5 | 6.41(3.32,10.28) | 9.20(4.77,14.86) | 0.44 | 1.03(0.54,1.66) | 1.4(0.73,2.26) | 0.48 (0.28, 0.67) |
| Global | 5-9 | 564.58(354.64,856.22) | 892.93(533.56,1380.70) | 0.58 | 96.75(60.77,146.73) | 129.97(77.66,200.96) | 0.64 (0.47, 0.81) |
| Global | 10-14 | 4947.89(3353.92,6849.78) | 8432.59(5583.09,11830.96) | 0.70 | 923.66(626.1,1278.7) | 1264.95(837.5,1774.73) | 0.66 (0.49, 0.83) |
| Global | 15-19 | 13814.77(10482.52,17853.46) | 21090.64(15607.28,27678.62) | 0.53 | 2659.63(2018.11,3437.17) | 3380.02(2501.25,4435.82) | 0.27 (0.10, 0.45) |
| Global | 20-24 | 19714.03(14989.84,26466.62) | 27965.58(21041.57,38573.57) | 0.42 | 4006.2(3046.17,5378.43) | 4683.11(3523.62,6459.52) | -0.08 (-0.28, 0.12) |
| Global | 25-29 | 19659.11(15964.56,24101.14) | 29282.84(23648.83,36802.14) | 0.49 | 4441.54(3606.83,5445.11) | 4977.17(4019.56,6255.22) | -0.14 (-0.32, 0.03) |
| Global | 30-34 | 18467.29(14667.26,22645.60) | 31383.93(24599.62,39151.55) | 0.70 | 4791.44(3805.5,5875.53) | 5191.89(4069.55,6476.9) | -0.18 (-0.33, -0.04) |
| Global | 35-39 | 18222.58(15082.46,21552.91) | 32113.54(26063.43,38451.40) | 0.76 | 5173.28(4281.82,6118.74) | 5725.71(4647.0,6855.72) | -0.12 (-0.27, 0.02) |
| Global | 40-44 | 15848.85(12679.73,19236.08) | 30617.21(24028.58,37684.90) | 0.93 | 5532.24(4426.02,6714.59) | 6120.37(4803.31,7533.2) | -0.13 (-0.27, 0.01) |
| Global | 45-49 | 13305.27(11328.90,15701.45) | 29476.92(24959.70,35040.85) | 1.22 | 5730.2(4879.03,6762.17) | 6225.26(5271.27,7400.32) | -0.12 (-0.23, -0.01) |
| Global | 50-54 | 12361.39(10755.26,14295.79) | 28093.55(24331.84,32602.78) | 1.27 | 5815.17(5059.59,6725.17) | 6314.25(5468.78,7327.73) | -0.06 (-0.15, 0.03) |
| Global | 55-59 | 10892.27(9172.20,12793.29) | 25419.50(21225.54,29961.38) | 1.33 | 5881.36(4952.59,6907.83) | 6423.48(5363.67,7571.21) | -0.02 (-0.11, 0.07) |
| Global | 60-64 | 9541.45(7830.14,11557.52) | 20657.38(16971.51,24852.10) | 1.17 | 5940.79(4875.27,7196.05) | 6454.47(5302.8,7765.12) | 0.01 (-0.07, 0.10) |
| Global | 65-69 | 7314.22(6171.20,8773.92) | 17593.58(14873.24,21109.04) | 1.41 | 5917.2(4992.5,7098.1) | 6378.13(5391.94,7652.58) | 0.05 (-0.02, 0.13) |
| Global | 70-74 | 4951.24(4145.86,5795.62) | 12651.14(10529.03,14972.46) | 1.56 | 5848.3(4897.0,6845.67) | 6146.13(5115.17,7273.86) | 0.07 (0.02, 0.12) |
| Global | 75-79 | 3527.25(2871.96,4267.55) | 7862.71(6358.78,9597.06) | 1.23 | 5730.19(4665.64,6932.85) | 5961.82(4821.48,7276.87) | 0.10 (0.08, 0.11) |
| Global | 80-84 | 2002.76(1621.90,2438.24) | 5020.82(4028.48,6178.37) | 1.51 | 5661.36(4584.77,6892.36) | 5732.64(4599.62,7054.3) | 0.07 (0.04, 0.09) |
| Global | 85-89 | 870.38(733.35,1055.87) | 2551.68(2150.56,3062.97) | 1.93 | 5759.9(4853.06,6987.39) | 5580.89(4703.57,6699.16) | -0.07 (-0.11, -0.03) |
| Global | 90-94 | 253.26(203.52,311.04) | 992.25(787.53,1213.13) | 2.92 | 5910.04(4749.31,7258.44) | 5546.57(4402.23,6781.28) | -0.16 (-0.21, -0.11) |
| Global | 95+ | 62.23(44.62,82.02) | 302.35(215.33,397.91) | 3.86 | 6112.85(4382.74,8055.97) | 5547.46(3950.81,7300.64) | -0.23 (-0.29, -0.18) |
| China | <5 | 0.91(0.46,1.47) | 0.76(0.39,1.22) | -0.17 | 0.81(0.41,1.31) | 0.97(0.51,1.57) | 0.37 (0.26, 0.49) |
| China | 5-9 | 62.15(40.41,89.95) | 58.11(37.26,84.10) | -0.06 | 59.6(38.75,86.26) | 60.68(38.91,87.82) | 0.39 (0.23, 0.56) |
| China | 10-14 | 600.82(414.14,808.33) | 435.05(301.63,583.10) | -0.28 | 587.36(404.86,790.21) | 504.74(349.95,676.51) | -0.14 (-0.34, 0.06) |
| China | 15-19 | 2379.53(1800.82,3018.54) | 1021.62(779.07,1315.93) | -0.57 | 1878.6(1421.72,2383.09) | 1368.14(1043.32,1762.28) | -1.02 (-1.12, -0.93) |
| China | 20-24 | 3987.01(3058.97,5176.97) | 1504.82(1155.55,1953.96) | -0.62 | 3020.44(2317.39,3921.92) | 2056.49(1579.18,2670.29) | -1.55 (-1.74, -1.35) |
| China | 25-29 | 3728.79(3008.60,4505.64) | 2145.44(1731.68,2598.61) | -0.42 | 3393.22(2737.85,4100.15) | 2480.79(2002.36,3004.79) | -1.27 (-1.47, -1.07) |
| China | 30-34 | 3326.60(2729.27,4010.90) | 3612.18(2996.02,4310.28) | 0.09 | 3769.76(3092.86,4545.22) | 2981.5(2472.92,3557.7) | -1.03 (-1.21, -0.85) |
| China | 35-39 | 3919.21(3305.19,4593.11) | 3784.12(3144.19,4520.00) | -0.03 | 4290.84(3618.6,5028.63) | 3571.17(2967.25,4265.63) | -0.89 (-1.03, -0.75) |
| China | 40-44 | 3193.06(2597.99,3872.45) | 3889.44(3139.89,4754.47) | 0.22 | 4759.06(3872.14,5771.64) | 4249.19(3430.31,5194.23) | -0.70 (-0.81, -0.59) |
| China | 45-49 | 2610.97(2210.81,3097.36) | 5476.28(4572.55,6499.62) | 1.10 | 5058.15(4282.95,6000.43) | 4963.93(4144.74,5891.53) | -0.40 (-0.48, -0.32) |
| China | 50-54 | 2519.87(2191.21,2944.30) | 6742.64(5769.74,7882.55) | 1.68 | 5281.54(4592.69,6171.13) | 5578.93(4773.94,6522.1) | -0.14 (-0.22, -0.06) |
| China | 55-59 | 2376.88(2018.20,2765.41) | 6761.25(5741.57,7912.53) | 1.84 | 5480.59(4653.54,6376.46) | 6149.82(5222.35,7196.98) | 0.06 (-0.05, 0.18) |
| China | 60-64 | 2011.15(1663.77,2433.11) | 4781.51(4016.45,5740.80) | 1.38 | 5691.28(4708.24,6885.36) | 6549.55(5501.6,7863.55) | 0.20 (0.06, 0.34) |
| China | 65-69 | 1593.42(1341.69,1920.44) | 5121.42(4370.12,6102.94) | 2.21 | 5840.59(4917.91,7039.28) | 6676.91(5697.42,7956.53) | 0.25 (0.11, 0.39) |
| China | 70-74 | 1094.75(921.72,1295.45) | 3533.42(2963.28,4161.80) | 2.23 | 5817.68(4898.17,6884.21) | 6629.75(5560.0,7808.77) | 0.29 (0.16, 0.42) |
| China | 75-79 | 650.74(538.82,775.68) | 2148.91(1782.92,2554.74) | 2.30 | 5717.91(4734.54,6815.78) | 6488.47(5383.39,7713.84) | 0.34 (0.22, 0.47) |
| China | 80-84 | 301.38(248.14,359.38) | 1257.11(1023.61,1519.05) | 3.17 | 5689.59(4684.45,6784.45) | 6351.67(5171.9,7675.13) | 0.38 (0.27, 0.48) |
| China | 85-89 | 99.97(85.12,118.81) | 606.24(516.37,713.41) | 5.06 | 5926.57(5046.26,7043.36) | 6364.18(5420.77,7489.25) | 0.29 (0.20, 0.38) |
| China | 90-94 | 19.43(15.98,23.34) | 191.01(155.68,229.27) | 8.83 | 6331.53(5207.32,7607.67) | 6514.79(5309.87,7819.44) | 0.18 (0.10, 0.26) |
| China | 95+ | 2.75(2.05,3.54) | 43.31(31.55,55.77) | 14.78 | 6779.95(5055.11,8754.26) | 6777.1(4936.25,8726.0) | 0.10 (0.02, 0.17) |

**Supplementary Table 6. DALYs of depressive disorders by age group (Both sexes, Global and China, 1990–2021)**

| Location | Age(years) | 1990,number(thousands)  (95% UI) | 2021,number(thousands)  (95% UI) | percentage change  (100%) | 1990_per100000  (95% UI) | 2021_per100000  (95% UI) | EAPC(95% CI) |
| --- | --- | --- | --- | --- | --- | --- | --- |
| Global | <5 | 1.16(0.50,2.19) | 1.75(0.75,3.42) | 0.50 | 0.19(0.08,0.35) | 0.27(0.11,0.52) | 0.54 (0.31, 0.77) |
| Global | 5-9 | 104.61(51.23,184.68) | 171.69(79.51,306.88) | 0.64 | 17.93(8.78,31.65) | 24.99(11.57,44.67) | 0.71 (0.51, 0.90) |
| Global | 10-14 | 926.88(553.47,1402.11) | 1629.55(958.15,2495.22) | 0.76 | 173.03(103.32,261.74) | 244.44(143.73,374.3) | 0.72 (0.53, 0.91) |
| Global | 15-19 | 2550.15(1624.37,3808.42) | 3979.12(2494.53,6048.54) | 0.56 | 490.96(312.73,733.2) | 637.7(399.78,969.35) | 0.28 (0.08, 0.48) |
| Global | 20-24 | 3548.06(2262.64,5269.86) | 5109.52(3217.18,7651.38) | 0.44 | 721.02(459.8,1070.92) | 855.64(538.75,1281.3) | -0.12 (-0.34, 0.10) |
| Global | 25-29 | 3401.92(2252.49,4968.97) | 5157.31(3365.03,7577.53) | 0.52 | 768.59(508.9,1122.63) | 876.58(571.95,1287.94) | -0.18 (-0.39, 0.02) |
| Global | 30-34 | 3115.51(2017.17,4621.76) | 5387.84(3420.06,8011.82) | 0.73 | 808.34(523.37,1199.14) | 891.32(565.78,1325.41) | -0.21 (-0.38, -0.04) |
| Global | 35-39 | 3022.60(1988.46,4224.77) | 5459.26(3559.90,7735.85) | 0.81 | 858.1(564.51,1199.39) | 973.36(634.72,1379.27) | -0.12 (-0.30, 0.05) |
| Global | 40-44 | 2599.94(1674.07,3727.69) | 5147.26(3254.70,7448.01) | 0.98 | 907.54(584.36,1301.2) | 1028.94(650.61,1488.86) | -0.13 (-0.29, 0.04) |
| Global | 45-49 | 2164.26(1448.45,2989.11) | 4873.12(3232.65,6757.60) | 1.25 | 932.08(623.81,1287.33) | 1029.16(682.71,1427.15) | -0.13 (-0.27, 0.00) |
| Global | 50-54 | 1993.13(1347.81,2720.21) | 4585.05(3083.19,6302.89) | 1.30 | 937.63(634.05,1279.67) | 1030.53(692.97,1416.62) | -0.08 (-0.18, 0.03) |
| Global | 55-59 | 1740.45(1163.84,2410.02) | 4108.53(2727.73,5639.38) | 1.36 | 939.77(628.42,1301.31) | 1038.22(689.29,1425.06) | -0.03 (-0.13, 0.08) |
| Global | 60-64 | 1515.97(1028.51,2103.82) | 3325.20(2243.11,4607.17) | 1.19 | 943.89(640.38,1309.9) | 1038.97(700.87,1439.52) | 0.00 (-0.10, 0.10) |
| Global | 65-69 | 1156.35(787.46,1572.54) | 2792.75(1912.54,3786.19) | 1.42 | 935.49(637.05,1272.19) | 1012.45(693.34,1372.59) | 0.04 (-0.03, 0.12) |
| Global | 70-74 | 780.19(526.45,1055.80) | 2000.82(1363.38,2710.35) | 1.56 | 921.54(621.83,1247.09) | 972.03(662.35,1316.73) | 0.07 (0.03, 0.11) |
| Global | 75-79 | 556.45(371.80,777.96) | 1236.58(841.61,1738.53) | 1.22 | 903.97(604.01,1263.84) | 937.62(638.14,1318.23) | 0.10 (0.08, 0.11) |
| Global | 80-84 | 314.61(214.10,445.62) | 783.40(531.64,1113.25) | 1.49 | 889.32(605.2,1259.67) | 894.47(607.01,1271.08) | 0.06 (0.02, 0.10) |
| Global | 85-89 | 134.83(95.04,186.00) | 389.85(275.34,540.31) | 1.89 | 892.28(628.92,1230.92) | 852.66(602.22,1181.74) | -0.10 (-0.15, -0.05) |
| Global | 90-94 | 38.41(26.23,54.79) | 147.97(101.55,211.54) | 2.85 | 896.42(612.1,1278.53) | 827.14(567.63,1182.51) | -0.21 (-0.26, -0.15) |
| Global | 95+ | 9.18(5.60,13.82) | 43.79(26.32,65.97) | 3.77 | 901.83(550.09,1357.09) | 803.42(482.94,1210.37) | -0.28 (-0.35, -0.22) |
| China | <5 | 0.16(0.07,0.30) | 0.13(0.06,0.25) | -0.17 | 0.14(0.06,0.27) | 0.17(0.07,0.32) | 0.36 (0.24, 0.48) |
| China | 5-9 | 11.05(5.70,19.48) | 10.22(5.12,17.71) | -0.08 | 10.6(5.46,18.68) | 10.67(5.35,18.5) | 0.37 (0.18, 0.56) |
| China | 10-14 | 111.25(68.09,170.88) | 78.05(45.36,119.35) | -0.30 | 108.76(66.56,167.05) | 90.56(52.62,138.47) | -0.19 (-0.43, 0.05) |
| China | 15-19 | 442.39(283.12,654.30) | 180.09(113.03,266.79) | -0.59 | 349.26(223.52,516.56) | 241.17(151.36,357.28) | -1.19 (-1.30, -1.08) |
| China | 20-24 | 721.86(466.86,1068.11) | 253.13(162.12,374.93) | -0.65 | 546.86(353.68,809.17) | 345.92(221.56,512.38) | -1.84 (-2.05, -1.63) |
| China | 25-29 | 635.72(413.33,936.38) | 337.21(222.05,488.39) | -0.47 | 578.51(376.13,852.11) | 389.92(256.76,564.73) | -1.58 (-1.80, -1.34) |
| China | 30-34 | 537.66(343.59,796.71) | 539.24(347.74,785.13) | 0.00 | 609.29(389.36,902.84) | 445.09(287.03,648.05) | -1.31 (-1.53, -1.09) |
| China | 35-39 | 607.55(405.60,843.02) | 546.08(355.66,762.80) | -0.10 | 665.16(444.06,922.96) | 515.35(335.64,719.87) | -1.12 (-1.31, -0.94) |
| China | 40-44 | 477.28(307.29,671.45) | 553.25(357.45,784.15) | 0.16 | 711.36(457.99,1000.75) | 604.43(390.51,856.68) | -0.85 (-0.99, -0.70) |
| China | 45-49 | 380.02(258.82,526.39) | 787.63(541.85,1076.19) | 1.07 | 736.2(501.4,1019.76) | 713.94(491.16,975.51) | -0.43 (-0.54, -0.32) |
| China | 50-54 | 359.46(249.75,488.83) | 981.90(683.77,1347.95) | 1.73 | 753.41(523.47,1024.57) | 812.43(565.76,1115.31) | -0.08 (-0.18, 0.01) |
| China | 55-59 | 332.74(226.57,451.08) | 991.15(678.23,1358.84) | 1.98 | 767.23(522.41,1040.1) | 901.51(616.89,1235.96) | 0.19 (0.08, 0.30) |
| China | 60-64 | 278.19(191.14,387.19) | 700.98(481.57,969.04) | 1.52 | 787.24(540.89,1095.69) | 960.18(659.63,1327.36) | 0.36 (0.23, 0.49) |
| China | 65-69 years | 222.01(153.82,302.77) | 752.42(529.08,1030.62) | 2.39 | 813.77(563.84,1109.78) | 980.95(689.77,1343.65) | 0.42 (0.30, 0.53) |
| China | 70-74 years | 154.43(106.92,207.63) | 523.22(363.32,711.48) | 2.39 | 820.66(568.2,1103.39) | 981.72(681.69,1334.96) | 0.45 (0.35, 0.55) |
| China | 75-79 years | 93.30(63.77,127.84) | 320.95(218.83,448.43) | 2.44 | 819.8(560.33,1123.32) | 969.09(660.73,1353.99) | 0.49 (0.40, 0.59) |
| China | 80-84 years | 43.81(30.30,60.61) | 187.80(129.91,261.60) | 3.29 | 827.11(572.01,1144.29) | 948.87(656.4,1321.77) | 0.49 (0.41, 0.57) |
| China | 85-89 years | 14.57(10.39,19.80) | 89.10(63.57,122.82) | 5.12 | 863.69(615.96,1173.56) | 935.32(667.36,1289.31) | 0.35 (0.29, 0.41) |
| China | 90-94 years | 2.81(1.93,3.98) | 27.38(19.17,38.57) | 8.74 | 916.11(628.91,1295.56) | 933.96(653.92,1315.37) | 0.20 (0.14, 0.25) |
| China | 95+ years | 0.39(0.24,0.58) | 6.00(3.64,8.91) | 14.46 | 959.12(599.64,1430.54) | 939.2(569.19,1393.85) | 0.08 (0.02, 0.13) |

**Supplementary Table 7. Incidence of depressive disorders by age group (Female, Global and China, 1990–2021)**

| Location | Age  (years) | 1990,number(thousands)  (95% UI) | 2021,number(thousands)  (95% UI) | percentage change  (100%) | 1990_per100000  (95% UI) | 2021_per100000  (95% UI) | EAPC(95% CI) |
| --- | --- | --- | --- | --- | --- | --- | --- |
| Global | <5 | 8.22(4.30,13.44) | 12.30(6.22,20.48) | 0.50 | 2.74(1.43,4.48) | 3.87(1.96,6.44) | 0.54 (0.31, 0.77) |
| Global | 5-9 | 553.25(302.11,872.06) | 916.20(477.06,1462.36) | 0.66 | 194.92(106.44,307.25) | 275.48(143.44,439.71) | 0.74 (0.54, 0.95) |
| Global | 10-14 | 4700.41(2997.00,6833.08) | 8387.21(5392.38,12282.26) | 0.78 | 1797.96(1146.38,2613.73) | 2597.25(1669.85,3803.43) | 0.76 (0.56, 0.96) |
| Global | 15-19 | 11271.90(7961.27,15282.26) | 16955.63(11658.05,22811.61) | 0.50 | 4411.03(3115.48,5980.4) | 5583.94(3839.3,7512.47) | 0.13 (-0.09, 0.35) |
| Global | 20-24 | 13959.87(10428.25,19333.53) | 19524.17(14284.98,28189.08) | 0.40 | 5718.1(4271.51,7919.2) | 6646.48(4862.94,9596.22) | -0.27 (-0.53, -0.01) |
| Global | 25-29 | 12739.83(9669.20,17153.13) | 19103.09(13908.91,26658.74) | 0.50 | 5788.21(4393.1,7793.35) | 6564.92(4779.9,9161.48) | -0.31 (-0.56, -0.06) |
| Global | 30-34 | 11444.73(8533.87,14979.48) | 19955.62(14600.13,26940.78) | 0.74 | 6020.08(4488.94,7879.42) | 6675.66(4884.12,9012.38) | -0.28 (-0.48, -0.08) |
| Global | 35-39 | 11073.55(8646.65,14175.90) | 20443.43(15402.04,26638.88) | 0.85 | 6384.17(4985.0,8172.75) | 7358.97(5544.24,9589.13) | -0.15 (-0.35, 0.05) |
| Global | 40-44 | 9414.69(7254.62,12205.50) | 19207.16(14510.51,24950.93) | 1.04 | 6713.97(5173.54,8704.2) | 7742.02(5848.89,10057.21) | -0.14 (-0.35, 0.06) |
| Global | 45-49 | 7818.28(6366.62,9409.55) | 18059.50(14667.83,21686.44) | 1.31 | 6870.15(5594.54,8268.45) | 7663.94(6224.61,9203.11) | -0.17 (-0.33, -0.01) |
| Global | 50-54 | 7260.94(5970.10,8709.89) | 17045.85(13896.70,20688.23) | 1.35 | 6920.66(5690.32,8301.71) | 7645.85(6233.31,9279.62) | -0.13 (-0.26, -0.01) |
| Global | 55-59 | 6438.63(5001.43,8206.89) | 15550.19(11896.61,19789.24) | 1.42 | 6974.86(5417.97,8890.39) | 7736.28(5918.61,9845.22) | -0.09 (-0.22, 0.03) |
| Global | 60-64 | 5798.38(4520.88,7372.71) | 12880.90(9964.19,16541.86) | 1.22 | 7065.76(5509.03,8984.2) | 7829.81(6056.85,10055.17) | -0.07 (-0.19, 0.05) |
| Global | 65-69 | 4668.10(3854.78,5772.86) | 11051.30(9102.53,13674.76) | 1.37 | 7043.19(5816.06,8710.03) | 7674.04(6320.81,9495.77) | 0.01 (-0.08, 0.09) |
| Global | 70-74 | 3307.41(2638.78,4095.19) | 8264.31(6511.64,10309.87) | 1.50 | 7030.65(5609.33,8705.26) | 7550.92(5949.54,9419.89) | 0.09 (0.05, 0.14) |
| Global | 75-79 | 2578.22(1892.10,3366.73) | 5381.28(3904.90,7105.33) | 1.09 | 7098.11(5209.14,9268.96) | 7463.88(5416.12,9855.14) | 0.15 (0.11, 0.19) |
| Global | 80-84 | 1590.12(1194.01,2014.08) | 3723.04(2746.84,4808.93) | 1.34 | 7197.55(5404.58,9116.6) | 7309.93(5393.23,9442.02) | 0.09 (0.03, 0.16) |
| Global | 85-89 | 745.61(601.00,924.51) | 2025.05(1608.32,2545.14) | 1.72 | 7421.17(5981.85,9201.85) | 7113.13(5649.34,8939.98) | -0.09 (-0.16, -0.01) |
| Global | 90-94 | 229.58(174.96,303.34) | 841.94(633.34,1113.37) | 2.67 | 7586.35(5781.56,10023.84) | 6980.72(5251.23,9231.29) | -0.22 (-0.29, -0.15) |
| Global | 95+ | 58.83(39.31,83.71) | 270.10(172.09,386.29) | 3.59 | 7762.1(5187.51,11045.19) | 6858.32(4369.79,9808.57) | -0.31 (-0.38, -0.24) |
| China | <5 | 1.15(0.58,1.88) | 0.96(0.50,1.59) | -0.17 | 2.19(1.1,3.59) | 2.66(1.38,4.4) | 0.44 (0.31, 0.57) |
| China | 5-9 | 57.83(31.91,91.15) | 53.11(29.56,83.77) | -0.08 | 115.57(63.76,182.16) | 118.48(65.93,186.87) | 0.52 (0.29, 0.75) |
| China | 10-14 | 586.34(380.46,827.32) | 381.16(244.59,547.96) | -0.35 | 1184.69(768.72,1671.59) | 948.16(608.44,1363.08) | -0.13 (-0.40, 0.13) |
| China | 15-19 | 2097.84(1503.89,2880.93) | 705.38(503.03,936.46) | -0.66 | 3403.72(2440.05,4674.26) | 2039.29(1454.31,2707.38) | -1.57 (-1.73, -1.41) |
| China | 20-24 | 2986.03(2227.75,4077.22) | 856.82(628.18,1185.18) | -0.71 | 4630.44(3454.57,6322.55) | 2497.54(1831.08,3454.66) | -2.26 (-2.57, -1.95) |
| China | 25-29 | 2411.70(1838.88,3130.28) | 1049.96(785.23,1389.29) | -0.56 | 4510.98(3439.53,5855.05) | 2569.26(1921.47,3399.59) | -2.01 (-2.34, -1.69) |
| China | 30-34 | 1910.70(1436.86,2466.81) | 1635.23(1232.94,2129.28) | -0.14 | 4525.63(3403.31,5842.82) | 2796.72(2108.69,3641.69) | -1.69 (-2.00, -1.38) |
| China | 35-39 | 2104.56(1640.71,2632.05) | 1642.00(1220.89,2114.70) | -0.22 | 4770.84(3719.33,5966.6) | 3182.35(2366.19,4098.49) | -1.40 (-1.67, -1.12) |
| China | 40-44 | 1555.13(1202.81,1987.96) | 1671.44(1250.84,2157.67) | 0.07 | 4872.84(3768.86,6229.05) | 3746.92(2804.04,4836.92) | -0.92 (-1.16, -0.69) |
| China | 45-49 | 1174.92(966.32,1423.39) | 2435.68(1987.12,2929.83) | 1.07 | 4819.17(3963.54,5838.32) | 4488.89(3662.21,5399.61) | -0.35 (-0.55, -0.16) |
| China | 50-54 | 1071.16(880.09,1273.48) | 3111.44(2513.69,3786.96) | 1.90 | 4770.2(3919.3,5671.17) | 5210.23(4209.26,6341.41) | 0.10 (-0.07, 0.27) |
| China | 55-59 | 976.02(765.72,1244.77) | 3250.48(2510.43,4059.93) | 2.33 | 4728.68(3709.8,6030.78) | 5903.66(4559.54,7373.81) | 0.47 (0.31, 0.64) |
| China | 60-64 | 832.21(649.30,1074.76) | 2320.22(1824.15,2905.11) | 1.79 | 4856.93(3789.42,6272.47) | 6377.76(5014.18,7985.5) | 0.67 (0.50, 0.84) |
| China | 65-69 | 720.10(595.56,884.37) | 2617.26(2165.01,3196.80) | 2.63 | 5190.56(4292.87,6374.68) | 6717.22(5556.52,8204.61) | 0.73 (0.57, 0.88) |
| China | 70-74 | 555.93(440.96,693.02) | 1941.76(1541.23,2428.21) | 2.49 | 5508.44(4369.29,6866.8) | 7076.4(5616.72,8849.18) | 0.77 (0.62, 0.92) |
| China | 75-79 | 376.65(278.64,501.77) | 1293.34(955.43,1698.31) | 2.43 | 5809.16(4297.58,7738.92) | 7383.55(5454.45,9695.49) | 0.81 (0.66, 0.96) |
| China | 80-84 | 202.01(157.09,258.04) | 837.12(627.69,1067.19) | 3.14 | 6201.66(4822.6,7921.87) | 7530.13(5646.3,9599.71) | 0.72 (0.60, 0.85) |
| China | 85-89 | 76.24(62.10,94.38) | 456.01(363.77,567.48) | 4.98 | 6697.82(5456.19,8291.71) | 7543.77(6017.88,9387.86) | 0.50 (0.41, 0.59) |
| China | 90-94 | 16.31(12.52,21.38) | 159.67(121.71,206.35) | 8.79 | 7236.93(5554.35,9489.07) | 7520.29(5732.63,9718.95) | 0.26 (0.20, 0.33) |
| China | 95+ | 2.50(1.73,3.53) | 39.17(25.54,55.06) | 14.68 | 7634.79(5295.24,10785.16) | 7559.57(4929.32,10625.45) | 0.09 (0.04, 0.14) |

**Supplementary Table 8. Prevalence of depressive disorders by age group (Female, Global and China, 1990–2021)**

| Location | Age  (years) | 1990,number(thousands)  (95% UI) | 2021,number(thousands)  (95% UI) | Percentage  change  (100%) | 1990_per100000  (95% UI) | 2021_per100000  (95% UI) | EAPC(95% CI) |
| --- | --- | --- | --- | --- | --- | --- | --- |
| Global | <5 | 3.39(1.78,5.40) | 4.88(2.54,7.77) | 0.44 | 1.13(0.59,1.8) | 1.53(0.8,2.44) | 0.49 (0.29, 0.69) |
| Global | 5-9 | 327.32(204.50,491.73) | 516.65(306.87,793.83) | 0.58 | 115.32(72.05,173.25) | 155.35(92.27,238.69) | 0.65 (0.48, 0.83) |
| Global | 10-14 | 3051.00(2063.06,4228.54) | 5195.21(3473.20,7266.16) | 0.70 | 1167.04(789.15,1617.46) | 1608.79(1075.54,2250.1) | 0.69 (0.52, 0.86) |
| Global | 15-19 | 8599.33(6529.06,11098.07) | 12840.70(9489.52,16836.97) | 0.49 | 3365.18(2555.02,4343.01) | 4228.78(3125.15,5544.86) | 0.26 (0.08, 0.43) |
| Global | 20-24 | 12186.73(9264.41,16382.32) | 16718.17(12607.12,23077.96) | 0.37 | 4991.8(3794.79,6710.35) | 5691.25(4291.75,7856.27) | -0.15 (-0.35, 0.06) |
| Global | 25-29 | 12010.97(9765.94,14816.69) | 17500.01(14180.52,22147.97) | 0.46 | 5457.06(4437.05,6731.81) | 6014.01(4873.24,7611.32) | -0.19 (-0.37, -0.01) |
| Global | 30-34 | 11130.37(8817.95,13741.86) | 18739.69(14686.00,23575.26) | 0.68 | 5854.73(4638.37,7228.41) | 6268.9(4912.84,7886.52) | -0.21 (-0.36, -0.07) |
| Global | 35-39 | 10993.55(9079.52,13008.17) | 19275.72(15606.83,23134.15) | 0.75 | 6338.04(5234.56,7499.52) | 6938.64(5617.95,8327.55) | -0.15 (-0.30, -0.00) |
| Global | 40-44 | 9492.70(7595.90,11510.35) | 18420.97(14521.67,22726.53) | 0.94 | 6769.6(5416.92,8208.46) | 7425.12(5853.39,9160.6) | -0.15 (-0.28, -0.01) |
| Global | 45-49 | 7932.18(6739.18,9375.16) | 17742.68(14956.26,21023.65) | 1.24 | 6970.24(5921.91,8238.23) | 7529.49(6347.01,8921.84) | -0.14 (-0.25, -0.03) |
| Global | 50-54 | 7370.12(6402.29,8479.19) | 16956.27(14607.85,19659.67) | 1.30 | 7024.72(6102.26,8081.82) | 7605.67(6552.29,8818.27) | -0.08 (-0.17, 0.01) |
| Global | 55-59 | 6522.88(5495.33,7651.12) | 15441.30(12860.67,18218.78) | 1.37 | 7066.13(5953.0,8288.33) | 7682.1(6398.23,9063.91) | -0.05 (-0.14, 0.05) |
| Global | 60-64 | 5783.02(4744.12,6958.02) | 12552.07(10233.58,15090.82) | 1.17 | 7047.04(5781.07,8478.86) | 7629.93(6220.61,9173.14) | -0.01 (-0.10, 0.08) |
| Global | 65-69 | 4556.34(3878.10,5452.53) | 10727.98(9043.05,12798.78) | 1.35 | 6874.56(5851.24,8226.73) | 7449.52(6279.51,8887.49) | 0.05 (-0.03, 0.13) |
| Global | 70-74 | 3133.81(2635.56,3664.64) | 7756.79(6495.45,9121.06) | 1.48 | 6661.65(5602.49,7790.04) | 7087.21(5934.74,8333.7) | 0.10 (0.05, 0.16) |
| Global | 75-79 | 2330.31(1904.05,2817.35) | 4884.59(3947.34,5957.12) | 1.10 | 6415.57(5242.04,7756.44) | 6774.96(5474.99,8262.57) | 0.15 (0.13, 0.17) |
| Global | 80-84 | 1378.09(1116.52,1678.01) | 3269.53(2627.97,4034.13) | 1.37 | 6237.83(5053.85,7595.41) | 6419.49(5159.84,7920.74) | 0.13 (0.10, 0.16) |
| Global | 85-89 | 630.92(530.28,763.78) | 1758.46(1477.71,2117.16) | 1.79 | 6279.69(5277.94,7602.04) | 6176.73(5190.58,7436.7) | -0.01 (-0.06, 0.03) |
| Global | 90-94 | 192.82(154.81,236.78) | 729.50(579.84,892.41) | 2.78 | 6371.7(5115.51,7824.4) | 6048.49(4807.64,7399.25) | -0.13 (-0.18, -0.07) |
| Global | 95+ | 49.53(35.58,65.42) | 235.09(167.86,310.61) | 3.75 | 6536.07(4694.32,8631.88) | 5969.47(4262.19,7887.09) | -0.21 (-0.28, -0.15) |
| China | <5 | 0.49(0.24,0.77) | 0.40(0.21,0.64) | -0.17 | 0.93(0.47,1.47) | 1.12(0.59,1.79) | 0.46 (0.33, 0.59) |
| China | 5-9 | 37.10(23.60,53.35) | 34.75(22.25,50.19) | -0.06 | 74.15(47.17,106.61) | 77.52(49.64,111.95) | 0.56 (0.37, 0.76) |
| China | 10-14 | 388.55(268.17,524.48) | 272.21(189.04,364.97) | -0.30 | 785.07(541.83,1059.7) | 677.14(470.24,907.87) | 0.04 (-0.18, 0.25) |
| China | 15-19 | 1569.53(1190.46,1985.30) | 619.23(474.86,788.97) | -0.61 | 2546.55(1931.51,3221.12) | 1790.23(1372.86,2280.97) | -0.96 (-1.08, -0.83) |
| China | 20-24 | 2607.40(1998.02,3412.53) | 885.12(682.22,1143.83) | -0.66 | 4043.3(3098.33,5291.82) | 2580.04(1988.59,3334.13) | -1.64 (-1.90, -1.37) |
| China | 25-29 | 2372.93(1912.66,2865.85) | 1255.32(1015.02,1518.89) | -0.47 | 4438.45(3577.55,5360.45) | 3071.76(2483.75,3716.73) | -1.35 (-1.61, -1.10) |
| China | 30-34 | 2062.94(1682.93,2487.11) | 2154.71(1774.13,2570.51) | 0.04 | 4886.22(3986.15,5890.9) | 3685.19(3034.28,4396.32) | -1.08 (-1.29, -0.87) |
| China | 35-39 | 2451.59(2079.00,2876.30) | 2305.92(1914.75,2772.11) | -0.06 | 5557.54(4712.89,6520.31) | 4469.09(3710.96,5372.61) | -0.90 (-1.06, -0.74) |
| China | 40-44 | 1969.69(1594.48,2386.72) | 2387.00(1922.45,2899.69) | 0.21 | 6171.82(4996.13,7478.54) | 5351.01(4309.63,6500.32) | -0.69 (-0.81, -0.58) |
| China | 45-49 | 1584.34(1345.65,1883.84) | 3368.63(2813.08,4023.77) | 1.13 | 6498.48(5519.47,7726.96) | 6208.3(5184.43,7415.71) | -0.40 (-0.47, -0.33) |
| China | 50-54 | 1503.25(1297.47,1758.97) | 4128.40(3527.03,4821.95) | 1.75 | 6694.43(5778.01,7833.19) | 6913.17(5906.14,8074.53) | -0.15 (-0.22, -0.08) |
| China | 55-59 | 1409.28(1192.40,1635.22) | 4142.68(3519.71,4804.56) | 1.94 | 6827.81(5777.02,7922.44) | 7524.1(6392.64,8726.24) | 0.06 (-0.04, 0.17) |
| China | 60-64 | 1192.27(982.88,1441.41) | 2886.11(2408.93,3432.01) | 1.42 | 6958.29(5736.23,8412.32) | 7933.28(6621.61,9433.83) | 0.22 (0.07, 0.36) |
| China | 65-69 | 971.24(816.06,1166.92) | 3114.74(2638.39,3702.72) | 2.21 | 7000.8(5882.29,8411.29) | 7994.0(6771.44,9503.07) | 0.29 (0.14, 0.44) |
| China | 70-74 | 691.46(579.49,813.66) | 2163.29(1819.32,2534.69) | 2.13 | 6851.37(5741.87,8062.15) | 7883.73(6630.18,9237.23) | 0.36 (0.21, 0.51) |
| China | 75-79 | 429.00(354.95,511.49) | 1341.76(1102.47,1596.36) | 2.13 | 6616.7(5474.61,7888.91) | 7659.98(6293.92,9113.45) | 0.44 (0.30, 0.59) |
| China | 80-84 | 210.26(173.60,250.20) | 821.34(668.66,992.93) | 2.91 | 6455.14(5329.54,7681.27) | 7388.25(6014.86,8931.7) | 0.46 (0.33, 0.59) |
| China | 85-89 | 74.79(63.38,89.20) | 434.39(370.50,511.88) | 4.81 | 6570.44(5567.89,7836.99) | 7186.06(6129.16,8468.03) | 0.34 (0.24, 0.44) |
| China | 90-94 | 15.49(12.72,18.77) | 150.92(123.11,181.73) | 8.74 | 6874.9(5644.4,8328.52) | 7108.5(5798.56,8559.64) | 0.19 (0.11, 0.27) |
| China | 95+ | 2.35(1.75,3.04) | 37.12(26.99,47.94) | 14.79 | 7184.11(5360.66,9292.34) | 7163.74(5208.99,9251.3) | 0.07 (0.01, 0.14) |

**Supplementary Table 9. DALYs of depressive disorders by age group (Female, Global and China, 1990–2021)**

| Location | Age  (years) | 1990,number(thousands)  (95% UI) | 2021,number(thousands)  (95% UI) | percentage  Change  (100%) | 1990_per100000  (95% UI) | 2021_per100000  (95% UI) | EAPC  (95% CI) |
| --- | --- | --- | --- | --- | --- | --- | --- |
| Global | <5 | 0.62(0.27,1.16) | 0.93(0.40,1.81) | 0.51 | 0.21(0.09,0.39) | 0.29(0.13,0.57) | 0.55 (0.32, 0.78) |
| Global | 5-9 | 61.05(29.90,106.40) | 99.79(46.80,178.23) | 0.63 | 21.51(10.53,37.49) | 30.0(14.07,53.59) | 0.72 (0.52, 0.92) |
| Global | 10-14 | 575.44(342.95,868.54) | 1008.09(593.67,1538.41) | 0.75 | 220.11(131.18,332.22) | 312.17(183.84,476.4) | 0.74 (0.55, 0.93) |
| Global | 15-19 | 1593.58(1017.79,2374.13) | 2421.17(1522.83,3649.93) | 0.52 | 623.61(398.29,929.07) | 797.36(501.51,1202.02) | 0.25 (0.06, 0.45) |
| Global | 20-24 | 2198.16(1403.68,3266.21) | 3040.49(1921.34,4552.66) | 0.38 | 900.39(574.96,1337.87) | 1035.05(654.07,1549.83) | -0.20 (-0.43, 0.03) |
| Global | 25-29 | 2081.76(1375.57,3019.81) | 3068.10(1977.87,4465.91) | 0.47 | 945.83(624.98,1372.02) | 1054.38(679.71,1534.74) | -0.25 (-0.46, -0.04) |
| Global | 30-34 | 1881.03(1218.28,2771.01) | 3205.88(2042.40,4741.60) | 0.70 | 989.45(640.83,1457.59) | 1072.45(683.23,1586.19) | -0.26 (-0.43, -0.08) |
| Global | 35-39 | 1829.20(1198.31,2565.76) | 3271.98(2112.19,4667.62) | 0.79 | 1054.58(690.86,1479.22) | 1177.81(760.32,1680.19) | -0.16 (-0.33, 0.01) |
| Global | 40-44 | 1564.36(1005.03,2239.26) | 3097.08(1951.31,4452.88) | 0.98 | 1115.6(716.72,1596.9) | 1248.37(786.54,1794.87) | -0.15 (-0.32, 0.02) |
| Global | 45-49 | 1297.25(865.54,1787.26) | 2937.73(1940.26,4062.88) | 1.26 | 1139.93(760.58,1570.52) | 1246.69(823.39,1724.17) | -0.16 (-0.29, -0.03) |
| Global | 50-54 | 1197.62(811.49,1639.45) | 2776.97(1870.94,3796.45) | 1.32 | 1141.5(773.46,1562.62) | 1245.6(839.2,1702.88) | -0.10 (-0.21, 0.00) |
| Global | 55-59 | 1052.54(706.07,1460.11) | 2510.20(1665.76,3430.15) | 1.38 | 1140.2(764.88,1581.71) | 1248.83(828.72,1706.51) | -0.06 (-0.17, 0.04) |
| Global | 60-64 | 928.78(629.66,1290.22) | 2034.47(1366.79,2789.68) | 1.19 | 1131.79(767.28,1572.23) | 1236.68(830.82,1695.74) | -0.03 (-0.14, 0.07) |
| Global | 65-69 | 727.51(495.97,987.53) | 1714.07(1172.66,2317.74) | 1.36 | 1097.65(748.31,1489.97) | 1190.25(814.29,1609.44) | 0.03 (-0.05, 0.11) |
| Global | 70-74 | 497.50(337.30,675.75) | 1234.62(844.46,1671.25) | 1.48 | 1057.56(717.0,1436.47) | 1128.05(771.57,1526.98) | 0.09 (0.05, 0.14) |
| Global | 75-79 | 370.35(246.91,518.15) | 772.14(525.35,1080.77) | 1.08 | 1019.62(679.78,1426.52) | 1070.97(728.66,1499.04) | 0.14 (0.12, 0.16) |
| Global | 80-84 | 217.91(148.17,308.37) | 512.52(346.62,729.59) | 1.35 | 986.33(670.67,1395.81) | 1006.3(680.57,1432.5) | 0.11 (0.07, 0.16) |
| Global | 85-89 | 98.32(69.17,135.29) | 269.78(190.74,375.45) | 1.74 | 978.57(688.48,1346.52) | 947.61(669.97,1318.8) | -0.05 (-0.11, 0.01) |
| Global | 90-94 | 29.40(20.11,42.10) | 109.23(74.97,156.15) | 2.71 | 971.63(664.44,1391.16) | 905.68(621.56,1294.67) | -0.17 (-0.24, -0.11) |
| Global | 95+ | 7.35(4.50,11.06) | 34.20(20.55,51.51) | 3.66 | 969.35(593.31,1459.4) | 868.37(521.81,1308.01) | -0.27 (-0.33, -0.20) |
| China | <5 | 0.09(0.04,0.16) | 0.07(0.03,0.13) | -0.17 | 0.16(0.07,0.3) | 0.2(0.09,0.36) | 0.44 (0.31, 0.57) |
| China | 5-9 | 6.66(3.40,11.56) | 6.17(3.04,10.73) | -0.07 | 13.32(6.79,23.09) | 13.75(6.77,23.94) | 0.53 (0.31, 0.74) |
| China | 10-14 | 72.68(43.91,110.86) | 49.17(28.73,73.80) | -0.32 | 146.85(88.72,223.98) | 122.3(71.47,183.59) | -0.02 (-0.27, 0.22) |
| China | 15-19 | 294.38(188.95,436.21) | 109.02(68.53,161.08) | -0.63 | 477.63(306.57,707.74) | 315.2(198.13,465.68) | -1.14 (-1.27, -1.00) |
| China | 20-24 | 476.28(307.31,710.59) | 147.96(94.47,219.37) | -0.69 | 738.57(476.55,1101.92) | 431.29(275.37,639.45) | -1.96 (-2.24, -1.68) |
| China | 25-29 | 407.90(266.65,599.76) | 195.98(128.29,281.79) | -0.52 | 762.95(498.76,1121.83) | 479.56(313.92,689.54) | -1.68 (-1.97, -1.39) |
| China | 30-34 | 335.89(217.18,494.57) | 320.01(206.99,468.39) | -0.05 | 795.58(514.41,1171.42) | 547.31(354.01,801.08) | -1.36 (-1.62, -1.10) |
| China | 35-39 | 383.68(253.39,537.72) | 333.27(217.92,463.09) | -0.13 | 869.76(574.4,1218.95) | 645.9(422.34,897.52) | -1.12 (-1.32, -0.91) |
| China | 40-44 | 297.68(192.00,422.45) | 341.17(220.08,483.75) | 0.15 | 932.75(601.61,1323.7) | 764.82(493.36,1084.43) | -0.81 (-0.97, -0.65) |
| China | 45-49 | 232.99(158.94,321.78) | 487.11(337.59,665.30) | 1.09 | 955.64(651.92,1319.83) | 897.74(622.16,1226.14) | -0.41 (-0.52, -0.30) |
| China | 50-54 | 216.97(150.64,293.23) | 605.47(420.53,833.84) | 1.79 | 966.22(670.84,1305.82) | 1013.88(704.2,1396.29) | -0.07 (-0.16, 0.01) |
| China | 55-59 | 199.45(135.37,272.78) | 612.84(416.83,841.65) | 2.07 | 966.29(655.87,1321.57) | 1113.06(757.07,1528.63) | 0.21 (0.11, 0.31) |
| China | 60-64 | 166.74(114.42,232.05) | 427.92(293.59,589.12) | 1.57 | 973.15(667.78,1354.3) | 1176.26(807.01,1619.36) | 0.40 (0.27, 0.52) |
| China | 65-69 | 136.66(94.37,185.98) | 462.38(326.05,633.32) | 2.38 | 985.07(680.23,1340.59) | 1186.71(836.82,1625.41) | 0.47 (0.35, 0.59) |
| China | 70-74 | 98.27(67.62,132.14) | 323.30(224.96,436.02) | 2.29 | 973.72(670.02,1309.31) | 1178.21(819.83,1588.99) | 0.54 (0.43, 0.65) |
| China | 75-79 | 61.82(42.15,85.19) | 201.77(136.73,282.61) | 2.26 | 953.55(650.06,1313.93) | 1151.87(780.57,1613.37) | 0.60 (0.49, 0.71) |
| China | 80-84 | 30.68(21.20,42.61) | 123.18(84.87,171.75) | 3.02 | 941.76(650.85,1308.07) | 1108.06(763.4,1544.96) | 0.58 (0.49, 0.67) |
| China | 85-89 | 10.92(7.76,14.81) | 63.92(45.85,87.93) | 4.85 | 959.46(681.84,1301.07) | 1057.39(758.46,1454.67) | 0.40 (0.34, 0.47) |
| China | 90-94 | 2.24(1.54,3.18) | 21.60(15.12,30.34) | 8.63 | 995.69(683.55,1409.21) | 1017.29(712.18,1429.23) | 0.20 (0.15, 0.25) |
| China | 95+ | 0.33(0.21,0.50) | 5.13(3.11,7.55) | 14.42 | 1016.03(633.65,1521.66) | 989.28(600.27,1457.92) | 0.04 (-0.00, 0.08) |

**Supplementary Table 10. Incidence of depressive disorders by age group (Male, Global and China, 1990–2021)**

| Location | Age  (years) | 1990,number(thousands)  (95% UI) | 2021,number(thousands)  (95% UI) | percentage  Change  (100%) | 1990_per100000  (95% UI) | 2021_per100000  (95% UI) | EAPC(95% CI) |
| --- | --- | --- | --- | --- | --- | --- | --- |
| Global | <5 | 7.29(3.71,11.99) | 10.87(5.42,18.59) | 0.49 | 2.28(1.16,3.75) | 3.2(1.59,5.47) | 0.52 (0.30, 0.74) |
| Global | 5-9 | 388.99(212.60,631.19) | 652.16(341.14,1057.57) | 0.68 | 129.79(70.94,210.61) | 183.98(96.24,298.35) | 0.72 (0.52, 0.92) |
| Global | 10-14 | 2795.83(1732.97,4043.71) | 5036.89(3146.36,7396.17) | 0.80 | 1019.44(631.89,1474.45) | 1465.45(915.41,2151.87) | 0.72 (0.52, 0.93) |
| Global | 15-19 | 6657.16(4651.89,9130.19) | 10893.21(7360.85,14919.91) | 0.64 | 2522.75(1762.85,3459.92) | 3400.62(2297.9,4657.67) | 0.27 (0.05, 0.49) |
| Global | 20-24 | 8353.88(6184.60,11498.48) | 13012.57(9492.60,18414.61) | 0.56 | 3369.14(2494.26,4637.36) | 4288.83(3128.68,6069.29) | -0.02 (-0.27, 0.23) |
| Global | 25-29 | 7828.38(5948.40,10557.50) | 12647.48(9502.95,17374.50) | 0.62 | 3518.05(2673.2,4744.52) | 4253.31(3195.82,5843.0) | -0.13 (-0.38, 0.11) |
| Global | 30-34 | 7203.63(5355.35,9418.33) | 13083.57(9687.60,17355.44) | 0.82 | 3688.24(2741.92,4822.16) | 4281.98(3170.55,5680.08) | -0.18 (-0.38, 0.03) |
| Global | 35-39 | 6880.30(5315.35,8818.74) | 13077.39(9887.83,17047.24) | 0.90 | 3848.23(2972.94,4932.42) | 4619.95(3493.15,6022.41) | -0.08 (-0.29, 0.13) |
| Global | 40-44 | 5926.66(4547.47,7674.76) | 12158.70(9189.24,15822.17) | 1.05 | 4052.25(3109.25,5247.48) | 4821.8(3644.2,6274.63) | -0.10 (-0.31, 0.11) |
| Global | 45-49 | 4968.81(4061.91,5981.25) | 11396.59(9244.29,13689.45) | 1.29 | 4196.82(3430.81,5051.95) | 4791.26(3886.41,5755.2) | -0.13 (-0.30, 0.04) |
| Global | 50-54 | 4585.25(3745.68,5549.27) | 10629.65(8618.36,12899.76) | 1.32 | 4259.22(3479.35,5154.7) | 4788.55(3882.49,5811.22) | -0.08 (-0.21, 0.05) |
| Global | 55-59 | 3997.93(3090.27,5131.60) | 9478.35(7296.93,12098.59) | 1.37 | 4304.03(3326.87,5524.5) | 4867.57(3747.31,6213.18) | -0.00 (-0.13, 0.13) |
| Global | 60-64 | 3499.19(2712.67,4486.49) | 7841.05(6105.59,10106.97) | 1.24 | 4454.94(3453.6,5711.92) | 5041.28(3925.49,6498.12) | 0.04 (-0.08, 0.16) |
| Global | 65-69 | 2643.99(2166.83,3272.90) | 6694.30(5461.80,8311.63) | 1.53 | 4611.78(3779.5,5708.75) | 5077.85(4142.96,6304.65) | 0.09 (0.01, 0.16) |
| Global | 70-74 | 1825.08(1434.02,2306.78) | 4962.74(3901.71,6278.28) | 1.72 | 4851.54(3812.0,6132.02) | 5148.53(4047.78,6513.32) | 0.12 (0.09, 0.15) |
| Global | 75-79 | 1260.66(929.12,1662.45) | 3143.94(2267.88,4199.79) | 1.49 | 4996.12(3682.18,6588.43) | 5258.59(3793.28,7024.63) | 0.13 (0.10, 0.17) |
| Global | 80-84 | 684.23(512.25,875.72) | 1899.56(1407.88,2468.48) | 1.78 | 5151.02(3856.33,6592.6) | 5182.7(3841.24,6734.93) | 0.06 (0.01, 0.10) |
| Global | 85-89 | 267.03(214.51,335.79) | 865.62(689.19,1093.76) | 2.24 | 5273.09(4235.95,6630.75) | 5017.33(3994.7,6339.66) | -0.12 (-0.17, -0.06) |
| Global | 90-94 | 67.35(50.60,90.20) | 284.47(212.55,379.10) | 3.22 | 5349.75(4019.26,7164.97) | 4880.68(3646.72,6504.31) | -0.25 (-0.29, -0.20) |
| Global | 95+ | 13.96(8.93,20.31) | 71.37(45.43,103.78) | 4.11 | 5364.15(3432.27,7804.75) | 4720.13(3004.87,6863.67) | -0.29 (-0.35, -0.23) |
| China | <5 | 0.99(0.50,1.66) | 0.82(0.41,1.36) | -0.17 | 1.67(0.84,2.8) | 1.98(0.99,3.27) | 0.27 (0.15, 0.39) |
| China | 5-9 | 37.25(20.80,59.78) | 34.07(18.78,54.20) | -0.09 | 68.68(38.35,110.21) | 66.88(36.87,106.4) | 0.17 (-0.04, 0.38) |
| China | 10-14 | 301.73(188.19,432.13) | 220.20(138.90,322.97) | -0.27 | 571.46(356.43,818.45) | 478.78(302.0,702.23) | -0.42 (-0.70, -0.13) |
| China | 15-19 | 1023.60(725.92,1391.46) | 470.51(326.48,635.71) | -0.54 | 1574.0(1116.27,2139.67) | 1173.83(814.52,1585.99) | -1.40 (-1.57, -1.22) |
| China | 20-24 | 1494.62(1114.22,2034.70) | 612.97(454.96,842.36) | -0.59 | 2213.79(1650.35,3013.74) | 1577.06(1170.54,2167.27) | -1.81 (-2.03, -1.60) |
| China | 25-29 | 1299.63(992.45,1693.49) | 760.95(573.08,1003.88) | -0.41 | 2303.23(1758.83,3001.23) | 1668.18(1256.31,2200.72) | -1.69 (-1.93, -1.45) |
| China | 30-34 | 1088.49(819.92,1418.14) | 1107.66(829.96,1449.59) | 0.02 | 2365.02(1781.49,3081.25) | 1767.05(1324.04,2312.55) | -1.56 (-1.81, -1.32) |
| China | 35-39 | 1148.80(893.37,1439.21) | 1016.08(761.42,1298.05) | -0.12 | 2432.56(1891.69,3047.48) | 1868.96(1400.55,2387.62) | -1.50 (-1.72, -1.27) |
| China | 40-44 | 880.31(676.71,1138.22) | 1006.06(768.05,1290.04) | 0.14 | 2502.29(1923.56,3235.42) | 2143.95(1636.76,2749.13) | -1.13 (-1.33, -0.93) |
| China | 45-49 | 695.52(575.64,841.21) | 1452.62(1187.11,1753.00) | 1.09 | 2553.4(2113.29,3088.29) | 2591.11(2117.52,3126.93) | -0.55 (-0.74, -0.37) |
| China | 50-54 | 663.02(546.75,800.19) | 1857.60(1509.24,2256.66) | 1.80 | 2625.25(2164.86,3168.38) | 3038.22(2468.46,3690.9) | -0.10 (-0.27, 0.08) |
| China | 55-59 | 610.64(471.10,773.88) | 1909.25(1483.17,2405.51) | 2.13 | 2686.64(2072.72,3404.84) | 3478.73(2702.39,4382.93) | 0.26 (0.08, 0.45) |
| China | 60-64 | 519.09(408.51,666.08) | 1399.60(1117.70,1756.69) | 1.70 | 2851.67(2244.21,3659.2) | 3821.39(3051.71,4796.37) | 0.48 (0.31, 0.65) |
| China | 65-69 | 421.25(345.39,514.98) | 1549.74(1280.07,1903.43) | 2.68 | 3141.62(2575.88,3840.66) | 4106.36(3391.79,5043.54) | 0.57 (0.43, 0.71) |
| China | 70-74 | 298.73(235.42,377.56) | 1139.41(893.24,1450.37) | 2.81 | 3423.76(2698.12,4327.14) | 4406.67(3454.61,5609.31) | 0.65 (0.53, 0.77) |
| China | 75-79 | 181.19(133.86,242.27) | 727.39(523.77,970.26) | 3.01 | 3699.95(2733.42,4947.32) | 4662.02(3356.96,6218.68) | 0.71 (0.59, 0.84) |
| China | 80-84 | 82.28(63.86,106.53) | 419.55(316.55,539.51) | 4.10 | 4033.87(3130.76,5222.36) | 4836.31(3649.03,6219.14) | 0.66 (0.55, 0.77) |
| China | 85-89 | 24.27(19.70,30.22) | 171.11(136.62,212.37) | 6.05 | 4424.68(3590.15,5508.71) | 4915.53(3924.73,6101.02) | 0.48 (0.39, 0.56) |
| China | 90-94 | 3.91(3.02,5.16) | 40.20(30.59,52.44) | 9.28 | 4801.44(3705.52,6332.19) | 4970.6(3782.04,6483.39) | 0.32 (0.25, 0.40) |
| China | 95+ | 0.40(0.27,0.55) | 6.21(4.15,8.85) | 14.70 | 5091.33(3474.88,7130.26) | 5133.21(3431.19,7313.68) | 0.31 (0.24, 0.39) |

**Supplementary Table 11. Prevalence of depressive disorders by age group (Male, Global and China, 1990–2021)**

| Location | Age  (years) | 1990,number(thousands)  (95% UI) | 2021,number(thousands)  (95% UI) | percentage  Change  (100%) | 1990_per100000  (95% UI) | 2021_per100000  (95% UI) | EAPC(95% CI) |
| --- | --- | --- | --- | --- | --- | --- | --- |
| Global | <5 | 3.02(1.56,4.86) | 4.33(2.24,7.08) | 0.43 | 0.94(0.49,1.52) | 1.27(0.66,2.08) | 0.47 (0.28, 0.66) |
| Global | 5-9 | 237.26(150.14,361.66) | 376.28(228.13,585.47) | 0.59 | 79.17(50.1,120.67) | 106.15(64.36,165.16) | 0.62 (0.45, 0.78) |
| Global | 10-14 | 1896.89(1288.90,2613.84) | 3237.37(2141.34,4533.99) | 0.71 | 691.66(469.97,953.08) | 941.89(623.01,1319.13) | 0.64 (0.47, 0.80) |
| Global | 15-19 | 5215.43(3946.02,6759.55) | 8249.94(6063.65,10866.20) | 0.58 | 1976.41(1495.36,2561.55) | 2575.45(1892.94,3392.19) | 0.32 (0.15, 0.50) |
| Global | 20-24 | 7527.31(5704.86,10049.32) | 11247.41(8386.92,15390.11) | 0.49 | 3035.78(2300.78,4052.91) | 3707.05(2764.25,5072.45) | 0.04 (-0.15, 0.23) |
| Global | 25-29 | 7648.14(6179.42,9329.35) | 11782.84(9470.97,14606.20) | 0.54 | 3437.06(2777.02,4192.59) | 3962.54(3185.06,4912.03) | -0.06 (-0.24, 0.11) |
| Global | 30-34 | 7336.92(5855.11,8936.74) | 12644.23(9884.79,15674.21) | 0.72 | 3756.49(2997.8,4575.59) | 4138.2(3235.09,5129.85) | -0.14 (-0.29, 0.00) |
| Global | 35-39 | 7229.03(6007.13,8585.30) | 12837.82(10451.14,15405.68) | 0.78 | 4043.28(3359.86,4801.86) | 4535.32(3692.16,5442.49) | -0.10 (-0.25, 0.05) |
| Global | 40-44 | 6356.14(5083.10,7694.15) | 12196.23(9580.39,15000.55) | 0.92 | 4345.9(3475.48,5260.73) | 4836.69(3799.32,5948.8) | -0.12 (-0.27, 0.02) |
| Global | 45-49 | 5373.09(4543.54,6369.49) | 11734.23(9914.81,13964.53) | 1.18 | 4538.28(3837.61,5379.87) | 4933.21(4168.3,5870.85) | -0.12 (-0.24, -0.01) |
| Global | 50-54 | 4991.27(4330.85,5783.86) | 11137.28(9677.45,12933.13) | 1.23 | 4636.37(4022.91,5372.61) | 5017.24(4359.6,5826.25) | -0.07 (-0.16, 0.02) |
| Global | 55-59 | 4369.39(3681.49,5154.29) | 9978.20(8350.63,11832.43) | 1.28 | 4703.93(3963.37,5548.93) | 5124.27(4288.43,6076.5) | -0.01 (-0.09, 0.08) |
| Global | 60-64 | 3758.43(3087.63,4574.34) | 8105.31(6684.87,9783.03) | 1.16 | 4785.0(3930.98,5823.76) | 5211.18(4297.93,6289.85) | 0.04 (-0.05, 0.12) |
| Global | 65-69 | 2757.88(2305.02,3314.87) | 6865.60(5771.69,8263.32) | 1.49 | 4810.43(4020.52,5781.97) | 5207.79(4378.02,6268.01) | 0.08 (0.01, 0.16) |
| Global | 70-74 | 1817.42(1522.19,2142.90) | 4894.35(4030.99,5824.87) | 1.69 | 4831.19(4046.38,5696.39) | 5077.58(4181.9,6042.93) | 0.10 (0.06, 0.15) |
| Global | 75-79 | 1196.94(979.09,1448.98) | 2978.12(2415.53,3639.94) | 1.49 | 4743.59(3880.23,5742.44) | 4981.24(4040.24,6088.2) | 0.12 (0.11, 0.14) |
| Global | 80-84 | 624.67(504.31,761.81) | 1751.30(1406.67,2151.97) | 1.80 | 4702.59(3796.54,5735.03) | 4778.19(3837.93,5871.38) | 0.08 (0.05, 0.10) |
| Global | 85-89 | 239.46(201.42,288.97) | 793.22(667.03,952.07) | 2.31 | 4728.65(3977.5,5706.29) | 4597.67(3866.26,5518.42) | -0.06 (-0.10, -0.02) |
| Global | 90-94 | 60.43(48.36,74.54) | 262.75(207.90,322.42) | 3.35 | 4800.34(3841.56,5921.0) | 4507.95(3566.87,5531.77) | -0.17 (-0.20, -0.13) |
| Global | 95+ | 12.70(9.09,16.88) | 67.26(47.77,88.90) | 4.30 | 4880.33(3492.09,6485.19) | 4448.3(3159.49,5879.5) | -0.21 (-0.25, -0.16) |
| China | <5 | 0.42(0.21,0.69) | 0.35(0.17,0.58) | -0.17 | 0.71(0.36,1.16) | 0.84(0.42,1.38) | 0.28 (0.17, 0.39) |
| China | 5-9 | 25.04(16.40,36.83) | 23.36(15.14,33.93) | -0.07 | 46.17(30.23,67.91) | 45.85(29.71,66.6) | 0.20 (0.04, 0.36) |
| China | 10-14 | 212.27(145.42,290.48) | 162.84(112.37,218.52) | -0.23 | 402.03(275.42,550.15) | 354.05(244.33,475.13) | -0.28 (-0.51, -0.06) |
| China | 15-19 | 810.00(616.55,1038.76) | 402.39(306.18,522.81) | -0.50 | 1245.55(948.08,1597.32) | 1003.9(763.87,1304.32) | -0.98 (-1.13, -0.84) |
| China | 20-24 | 1379.61(1054.69,1793.36) | 619.70(467.78,799.18) | -0.55 | 2043.45(1562.19,2656.28) | 1594.39(1203.53,2056.15) | -1.36 (-1.52, -1.19) |
| China | 25-29 | 1355.86(1100.27,1642.28) | 890.12(721.60,1079.25) | -0.34 | 2402.87(1949.92,2910.48) | 1951.34(1581.91,2365.97) | -1.15 (-1.32, -0.99) |
| China | 30-34 | 1263.66(1031.88,1522.93) | 1457.47(1205.62,1744.89) | 0.15 | 2745.6(2242.0,3308.93) | 2325.11(1923.34,2783.64) | -0.99 (-1.14, -0.83) |
| China | 35-39 | 1467.62(1234.64,1734.54) | 1478.20(1218.65,1773.46) | 0.01 | 3107.64(2614.33,3672.83) | 2718.98(2241.57,3262.08) | -0.91 (-1.05, -0.77) |
| China | 40-44 | 1223.37(985.24,1490.47) | 1502.44(1204.83,1858.86) | 0.23 | 3477.45(2800.57,4236.7) | 3201.76(2567.54,3961.31) | -0.75 (-0.87, -0.63) |
| China | 45-49 | 1026.63(862.47,1239.06) | 2107.65(1766.88,2522.92) | 1.05 | 3768.99(3166.33,4548.88) | 3759.54(3151.68,4500.27) | -0.47 (-0.57, -0.37) |
| China | 50-54 | 1016.61(869.35,1205.30) | 2614.23(2233.09,3046.29) | 1.57 | 4025.3(3442.21,4772.4) | 4275.74(3652.37,4982.4) | -0.23 (-0.33, -0.13) |
| China | 55-59 | 967.60(821.08,1134.68) | 2618.57(2220.31,3063.78) | 1.71 | 4257.15(3612.5,4992.27) | 4771.14(4045.48,5582.33) | -0.04 (-0.17, 0.09) |
| China | 60-64 | 818.88(675.48,986.71) | 1895.40(1576.96,2281.05) | 1.31 | 4498.63(3710.81,5420.61) | 5175.1(4305.66,6228.07) | 0.10 (-0.05, 0.25) |
| China | 65-69 | 622.18(519.77,751.61) | 2006.69(1692.59,2424.31) | 2.23 | 4640.17(3876.43,5605.43) | 5317.13(4484.85,6423.71) | 0.17 (0.03, 0.32) |
| China | 70-74 | 403.29(339.05,481.41) | 1370.13(1151.50,1624.60) | 2.40 | 4622.05(3885.81,5517.39) | 5298.97(4453.44,6283.14) | 0.26 (0.13, 0.38) |
| China | 75-79 | 221.73(182.43,265.06) | 807.15(666.16,969.23) | 2.64 | 4527.93(3725.33,5412.61) | 5173.24(4269.57,6212.02) | 0.35 (0.25, 0.45) |
| China | 80-84 | 91.12(74.27,109.98) | 435.77(350.48,527.48) | 3.78 | 4467.13(3640.92,5391.4) | 5023.31(4040.15,6080.48) | 0.39 (0.32, 0.47) |
| China | 85-89 | 25.19(21.25,29.81) | 171.85(145.37,202.29) | 5.82 | 4590.71(3873.03,5433.34) | 4936.92(4176.18,5811.33) | 0.31 (0.25, 0.36) |
| China | 90-94 | 3.93(3.23,4.71) | 40.09(32.64,48.07) | 9.19 | 4828.75(3961.29,5782.69) | 4956.34(4035.51,5942.51) | 0.22 (0.18, 0.26) |
| China | 95+ | 0.39(0.30,0.50) | 6.19(4.57,7.97) | 14.70 | 5077.72(3816.97,6458.12) | 5120.61(3778.9,6586.43) | 0.22 (0.17, 0.27) |

**Supplementary Table 12. DALYs of depressive disorders by age group (Male, Global and China, 1990–2021)**

| Location | Age  (years) | 1990,number(thousands)  (95% UI) | 2021,number(thousands)  (95% UI) | percentage  change  (100%) | 1990_per100000  (95% UI) | 2021_per100000  (95% UI) | EAPC(95% CI) |
| --- | --- | --- | --- | --- | --- | --- | --- |
| Global | <5 | 0.55(0.23,1.03) | 0.82(0.35,1.61) | 0.50 | 0.17(0.07,0.32) | 0.24(0.1,0.47) | 0.53 (0.30, 0.75) |
| Global | 5-9 | 43.56(21.27,78.76) | 71.90(33.64,129.44) | 0.65 | 14.53(7.1,26.28) | 20.28(9.49,36.52) | 0.69 (0.50, 0.88) |
| Global | 10-14 | 351.44(207.29,534.60) | 621.47(362.51,957.61) | 0.77 | 128.15(75.58,194.93) | 180.81(105.47,278.61) | 0.70 (0.51, 0.89) |
| Global | 15-19 | 956.57(601.36,1448.62) | 1557.94(972.59,2375.71) | 0.63 | 362.5(227.89,548.96) | 486.36(303.62,741.64) | 0.35 (0.15, 0.54) |
| Global | 20-24 | 1349.90(859.27,2003.64) | 2069.03(1290.59,3108.48) | 0.53 | 544.42(346.55,808.07) | 681.93(425.37,1024.53) | 0.02 (-0.20, 0.24) |
| Global | 25-29 | 1320.16(874.85,1930.04) | 2089.21(1357.92,3107.43) | 0.58 | 593.28(393.16,867.36) | 702.6(456.66,1045.02) | -0.08 (-0.29, 0.12) |
| Global | 30-34 | 1234.48(796.57,1843.40) | 2181.97(1391.48,3248.61) | 0.77 | 632.05(407.84,943.81) | 714.11(455.4,1063.2) | -0.16 (-0.33, 0.02) |
| Global | 35-39 | 1193.40(790.69,1673.31) | 2187.28(1427.93,3089.00) | 0.83 | 667.48(442.24,935.9) | 772.72(504.46,1091.28) | -0.09 (-0.26, 0.09) |
| Global | 40-44 | 1035.58(668.30,1488.43) | 2050.18(1303.39,2978.40) | 0.98 | 708.06(456.93,1017.69) | 813.04(516.89,1181.15) | -0.11 (-0.29, 0.06) |
| Global | 45-49 | 867.01(581.41,1199.49) | 1935.39(1281.06,2683.76) | 1.23 | 732.3(491.08,1013.13) | 813.66(538.57,1128.28) | -0.13 (-0.27, 0.01) |
| Global | 50-54 | 795.51(535.68,1088.26) | 1808.08(1217.19,2489.36) | 1.27 | 738.95(497.59,1010.88) | 814.52(548.33,1121.43) | -0.07 (-0.18, 0.04) |
| Global | 55-59 | 687.91(460.00,949.91) | 1598.34(1060.79,2209.96) | 1.32 | 740.58(495.22,1022.64) | 820.82(544.76,1134.92) | -0.00 (-0.10, 0.10) |
| Global | 60-64 | 587.19(396.68,813.60) | 1290.73(870.69,1806.93) | 1.20 | 747.58(505.03,1035.82) | 829.85(559.8,1161.74) | 0.05 (-0.05, 0.15) |
| Global | 65-69 | 428.85(292.43,581.06) | 1078.69(737.18,1464.66) | 1.52 | 748.02(510.07,1013.52) | 818.22(559.18,1111.0) | 0.10 (0.02, 0.17) |
| Global | 70-74 | 282.68(189.93,382.74) | 766.20(520.22,1044.05) | 1.71 | 751.44(504.9,1017.43) | 794.88(539.7,1083.14) | 0.12 (0.09, 0.16) |
| Global | 75-79 | 186.09(125.65,258.70) | 464.44(315.13,655.44) | 1.50 | 737.5(497.95,1025.26) | 776.82(527.09,1096.29) | 0.14 (0.12, 0.16) |
| Global | 80-84 | 96.70(65.90,137.28) | 270.88(184.22,385.15) | 1.80 | 727.98(496.08,1033.49) | 739.07(502.63,1050.82) | 0.09 (0.05, 0.12) |
| Global | 85-89 | 36.52(25.82,50.50) | 120.07(84.95,166.19) | 2.29 | 721.07(509.96,997.27) | 695.96(492.39,963.26) | -0.07 (-0.12, -0.02) |
| Global | 90-94 | 9.01(6.11,12.84) | 38.74(26.53,55.80) | 3.30 | 715.65(484.97,1019.84) | 664.62(455.11,957.3) | -0.19 (-0.24, -0.15) |
| Global | 95+ | 1.84(1.11,2.78) | 9.59(5.78,14.46) | 4.23 | 705.19(425.0,1068.29) | 634.23(382.38,956.06) | -0.23 (-0.29, -0.17) |
| China | <5 | 0.07(0.03,0.14) | 0.06(0.02,0.12) | -0.17 | 0.12(0.05,0.23) | 0.15(0.06,0.28) | 0.27 (0.15, 0.39) |
| China | 5-9 | 4.39(2.19,7.76) | 4.06(2.00,7.11) | -0.08 | 8.09(4.04,14.3) | 7.96(3.92,13.95) | 0.19 (-0.00, 0.37) |
| China | 10-14 | 38.57(23.08,60.82) | 28.89(16.55,43.47) | -0.25 | 73.05(43.71,115.19) | 62.81(35.99,94.52) | -0.32 (-0.59, -0.06) |
| China | 15-19 | 148.01(92.78,223.10) | 71.06(44.76,105.88) | -0.52 | 227.6(142.68,343.06) | 177.29(111.67,264.15) | -1.12 (-1.29, -0.95) |
| China | 20-24 | 245.57(159.82,365.62) | 105.16(66.92,155.83) | -0.57 | 363.73(236.72,541.55) | 270.57(172.18,400.93) | -1.60 (-1.79, -1.41) |
| China | 25-29 | 227.83(147.01,336.24) | 141.23(92.95,206.47) | -0.38 | 403.76(260.54,595.89) | 309.62(203.78,452.63) | -1.43 (-1.63, -1.23) |
| China | 30-34 | 201.77(128.29,298.87) | 219.23(140.22,318.15) | 0.09 | 438.39(278.75,649.37) | 349.74(223.7,507.55) | -1.26 (-1.45, -1.06) |
| China | 35-39 | 223.88(150.28,311.80) | 212.81(139.34,301.72) | -0.05 | 474.05(318.21,660.22) | 391.44(256.29,554.98) | -1.17 (-1.34, -0.99) |
| China | 40-44 | 179.60(115.56,254.52) | 212.08(136.23,304.65) | 0.18 | 510.53(328.48,723.47) | 451.95(290.31,649.21) | -0.95 (-1.10, -0.80) |
| China | 45-49 | 147.03(98.87,203.71) | 300.51(206.74,412.87) | 1.04 | 539.79(362.97,747.88) | 536.04(368.78,736.46) | -0.55 (-0.68, -0.41) |
| China | 50-54 | 142.49(98.41,197.75) | 376.43(261.28,515.67) | 1.64 | 564.2(389.65,782.98) | 615.68(427.34,843.41) | -0.21 (-0.34, -0.09) |
| China | 55-59 | 133.29(91.26,182.59) | 378.31(258.83,519.82) | 1.84 | 586.46(401.52,803.34) | 689.29(471.59,947.14) | 0.05 (-0.09, 0.18) |
| China | 60-64 | 111.45(76.27,154.76) | 273.06(185.42,378.17) | 1.45 | 612.25(419.01,850.2) | 745.54(506.25,1032.54) | 0.23 (0.08, 0.38) |
| China | 65-69 | 85.35(59.37,116.72) | 290.04(205.43,395.98) | 2.40 | 636.54(442.74,870.47) | 768.52(544.33,1049.24) | 0.32 (0.19, 0.45) |
| China | 70-74 | 56.16(39.26,76.05) | 199.92(138.54,276.10) | 2.56 | 643.62(450.01,871.59) | 773.2(535.8,1067.81) | 0.41 (0.30, 0.51) |
| China | 75-79 | 31.47(21.48,42.99) | 119.18(80.57,168.10) | 2.79 | 642.72(438.53,877.97) | 763.89(516.38,1077.39) | 0.49 (0.40, 0.58) |
| China | 80-84 | 13.14(9.08,18.17) | 64.62(44.14,91.19) | 3.92 | 644.03(444.95,890.52) | 744.87(508.78,1051.13) | 0.51 (0.43, 0.58) |
| China | 85-89 | 3.65(2.63,4.98) | 25.18(17.75,35.17) | 5.90 | 665.0(479.48,907.4) | 723.33(509.93,1010.37) | 0.38 (0.32, 0.43) |
| China | 90-94 | 0.57(0.39,0.81) | 5.79(4.00,8.20) | 9.20 | 696.01(475.21,991.08) | 715.25(494.53,1013.37) | 0.27 (0.21, 0.32) |
| China | 95+ | 0.06(0.03,0.08) | 0.88(0.54,1.31) | 14.68 | 719.39(448.28,1050.05) | 724.65(443.27,1086.25) | 0.27 (0.20, 0.33) |

**Supplementary Table 13. Statistical Comparison of Sex-Specific Trends in Depressive Disorder Burden (1990–2021).**

| Location | Measures | Range | Lower Endpoint | Upper Endpoint | AAPC  Female | LowerCI  Female | UpperCI  Female | AAPC  Male | LowerCI  Male | UpperCI  Male | diff_F_minus_M | *z* | *p*_value | sig_0.05 |
| --- | --- | --- | --- | --- | --- | --- | --- | --- | --- | --- | --- | --- | --- | --- |
| Global | Prevalence | Full Range | 1990 | 2021 | 0.350 | 0.284 | 0.450 | 0.428 | 0.358 | 0.512 | -0.078 | -1.357 | 0.175 | No |
| Global | Dalys | Full Range | 1990 | 2021 | 0.401 | 0.320 | 0.526 | 0.469 | 0.395 | 0.573 | -0.068 | -0.982 | 0.326 | No |
| Global | Incidence | Full Range | 1990 | 2021 | 0.466 | 0.366 | 0.614 | 0.614 | 0.508 | 0.743 | -0.148 | -1.702 | 0.089 | No |
| China | Prevalence | Full Range | 1990 | 2021 | -0.315 | -0.345 | -0.288 | -0.100 | -0.144 | -0.067 | -0.215 | -8.814 | ＜0.001 | Yes |
| China | Incidence | Full Range | 1990 | 2021 | -0.446 | -0.541 | -0.354 | -0.093 | -0.141 | -0.031 | -0.353 | -6.377 | ＜0.001 | Yes |
| China | Dalys | Full Range | 1990 | 2021 | -0.368 | -0.426 | -0.297 | -0.160 | -0.190 | -0.124 | -0.208 | -5.659 | ＜0.001 | Yes |
| ***Note:*** *Z-test was used to assess the statistical differences between sex-specific trends in depressive disorder burden. A significance level of p < 0.05 was considered statistically significant. All values are presented to three decimal places for consistency.* | | | | | | | | | | | | | | |
